# Exploitation of functionalized green nanomaterials for plant disease management

**DOI:** 10.1186/s11671-024-04063-z

**Published:** 2024-07-18

**Authors:** Dhiraj L. Wasule, Prashant R. Shingote, Shreshtha Saxena

**Affiliations:** Vasantrao Naik College of Agricultural Biotechnology, Dr. Panjabrao Deshmukh Krishi Vidyapeeth, Akola, Maharashtra 444104 India

**Keywords:** Green synthesis, Crop management, Microorganism, Nanotechnology, Plant disease

## Abstract

A crucial determining factor in agricultural productivity is biotic stress. In addition, supply of quality food to the ever-increasing world’s population has raised the food demand tremendously. Therefore, enhanced agricultural crop productivity is the only option to mitigate these concerns. It ultimately demanded the often and indiscriminate use of synthetic agrochemicals such as chemical fertilizers, pesticides, insecticides, herbicides, etc. for the management of various biotic stresses including a variety of plant pathogens. However, the food chain and biosphere are severely impacted due to the use of such harmful agrochemicals and their byproducts. Hence, it is need of hour to search for novel, effective and ecofriendly approaches for the management of biotic stresses in crop plants. Particularly, in plant disease management, efforts are being made to take advantage of newly emerged science i.e. nanotechnology for the creation of inorganic nanoparticles (NPs) such as metallic, oxide, sulphide, etc. through different routes and their application in plant disease management. Among these, green nanomaterials which are synthesized using environmentally friendly methods and materials reported to possess unique properties (such as high surface area, adjustable size and shape, and specific functionalities) making them ideal candidates for targeted disease control. Nanotechnology can stop crop losses by managing specific diseases from soil, plants, and hydroponic systems. This review mainly focuses on the application of biologically produced green NPs in the treatment of plant diseases caused due to bacteria, viruses, and fungi. The utilization of green synthesis of NPs in the creation of intelligent targeted pesticide and biomolecule control delivery systems, for disease management is considered environmentally friendly due to its pursuit of less hazardous, sustainable, and environmentally friendly methods.

## Introduction

Crop and plant diseases have significant consequences on both production losses and food security. In recent times, the global capitalism and the environmental concerns have not only worsened the favourable conditions for plant but have also introduced new challenges that agriculture must overcome. According to the Food and Agriculture Organization of the United Nations (FAO), the worldwide annual economic losses due to plant diseases are of approximately US$220 billion. Among these, alone pests contributed for about 20–40% losses in crop production [1]. This implies that effective disease management is crucial to meet the rising food demand due to projected population growth by 2050 [2]. The rapidly growing arena of nanotechnology has opened new frontiers in agricultural sciences, particularly in plant disease management. The global nanotechnology industry was estimated to be worth $1.76 billion in 2020 and is projected to grow to $33.63 billion by 2030, with a compound annual growth rate (CAGR) of 36.4% from 2021 to 2030 [3]. Traditional approaches which are commonly used for combating plant diseases are often rely on chemical pesticides, which can pose environmental hazards and lead to the development of resistant pathogen strains. Using pesticides to control plant diseases puts both human health and the ecosystem at grave risk. In this context, nanotechnology offers innovative solutions that are more efficient, sustainable, and environmentally friendly. Among these, functionalized green nanomaterials have garnered significant attention due to their unique properties and potential for enhancing plant health and productivity [4]. Green synthesis method utilizes naturally occurring capping and stabilizing agents, avoiding hazardous chemicals and high-energy consumption. Nanotechnology, an innovative and rapidly emerging field, integrates knowledge from physics, chemistry, natural sciences, and other disciplines. The term “nanotechnology” refers to nanoparticles ranging in size from 1 to 100 nm (sizes ranging from 1 to 100 nm, or 1.0 × 10^–9^ m), with a high surface area-to-volume ratio that increase their reactivity [5].

**Fig. 1:**
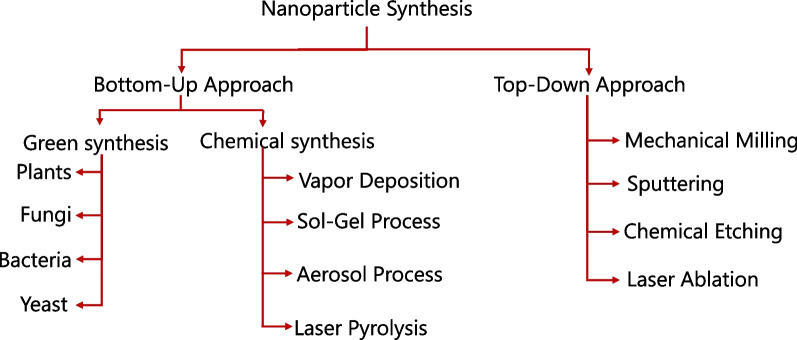
Various nanotechnological approaches used for fabrication of nanomaterials

The synthesis of nanoparticles (NPs) can be broadly categorized into two main approaches: top-down and bottom-up approach. Each approach has several techniques, which can be selected based on the desired properties and applications of the NPs. Top-down approaches involve breaking down bulk materials into nanoscale particles. Common techniques include: mechanical milling process, lithography, laser ablation, Electrospinning etc. However, bottom-up approach synthesized NPs from scratch, i.e. atom by atom. This is good for complex structures and functionalities which involves Chemical Vapor Deposition, Sol–Gel Process, Hydrothermal and Solvothermal Synthesis, Microemulsion, Co-precipitation, Green Synthesis, etc. The Fig. 1 illustrates two primary methods for fabricating nanomaterials: top-down and bottom-up methods (Fig. 1). The choice of synthesis method depends on the desired properties, application, and scale of production for the nanoparticles. Each method has its advantages and limitations in terms of cost, complexity, environmental impact, and control over particle size and morphology. A subset of the bottom-up approach is green synthesis, which emphasizes the use of environmentally friendly methods and materials. Techniques include: Plant Extracts: Using extracts from various plant parts (leaves, stems, roots) to reduce metal ions to nanoparticles. Microbial Synthesis: Utilizing bacteria, fungi, or yeast for the biosynthesis of nanoparticles. Enzyme-Mediated Synthesis: Using specific enzymes to catalyze the formation of nanoparticles.

Nanotechnology in agriculture includes nanobiotechnology, nanofertilizers, nanomicronutrients and nanoformulations for the control of plant diseases and insect pests. Biosensors and insecticides based on nanomaterials are developed using nanotechnology [6, 7]. Microorganisms are killed by these NPs because they interfere with their cell DNA, cellular metabolism, electron transport chain, and nutrition intake. Targets for NPs associated with biomolecules are specific. According to Elmer et al. [8], NPs used in plant protection include metalloids, metallic oxides, nanometals, carbon nanotubes, graphene oxides, and fullerenes. Biological, agricultural, and related sciences are among the diverse study areas that include environmentally friendly green and biosynthetic nanotechnology. Toxic chemicals or high energy inputs are not used in green synthesis. NPs produced via the green technique are being employed in agricultural and medicinal applications [9].

Present review explores the potential of functionalized, environmentally friendly nanomaterials for a specific purpose to combat plant diseases. It examines the synthesis methods for various NPs, including: Silver nanoparticles (AgNPs), Zinc- and iron-based nanoparticles (ZnONPS, Fe_3_O_4_NPs, Copper nanoparticles (CuNPs), Chitosan and its derivatives nanoparticles (Ch-CuNPs), Sulfur nanoparticles (SNPs) and their mode of action in disease management. The impact of these nanoparticles against bacteria, fungi, and viruses will be discussed followed by use of nano-fungicides with enhanced efficiency over conventional fungicides. Finally, the review highlights the prospects of nanotechnology in plant disease management along with its limitations and future directions.

## Biogenic green synthesis: a sustainable approach for synthesis of functionalized nanomaterials

**Fig. 2:**
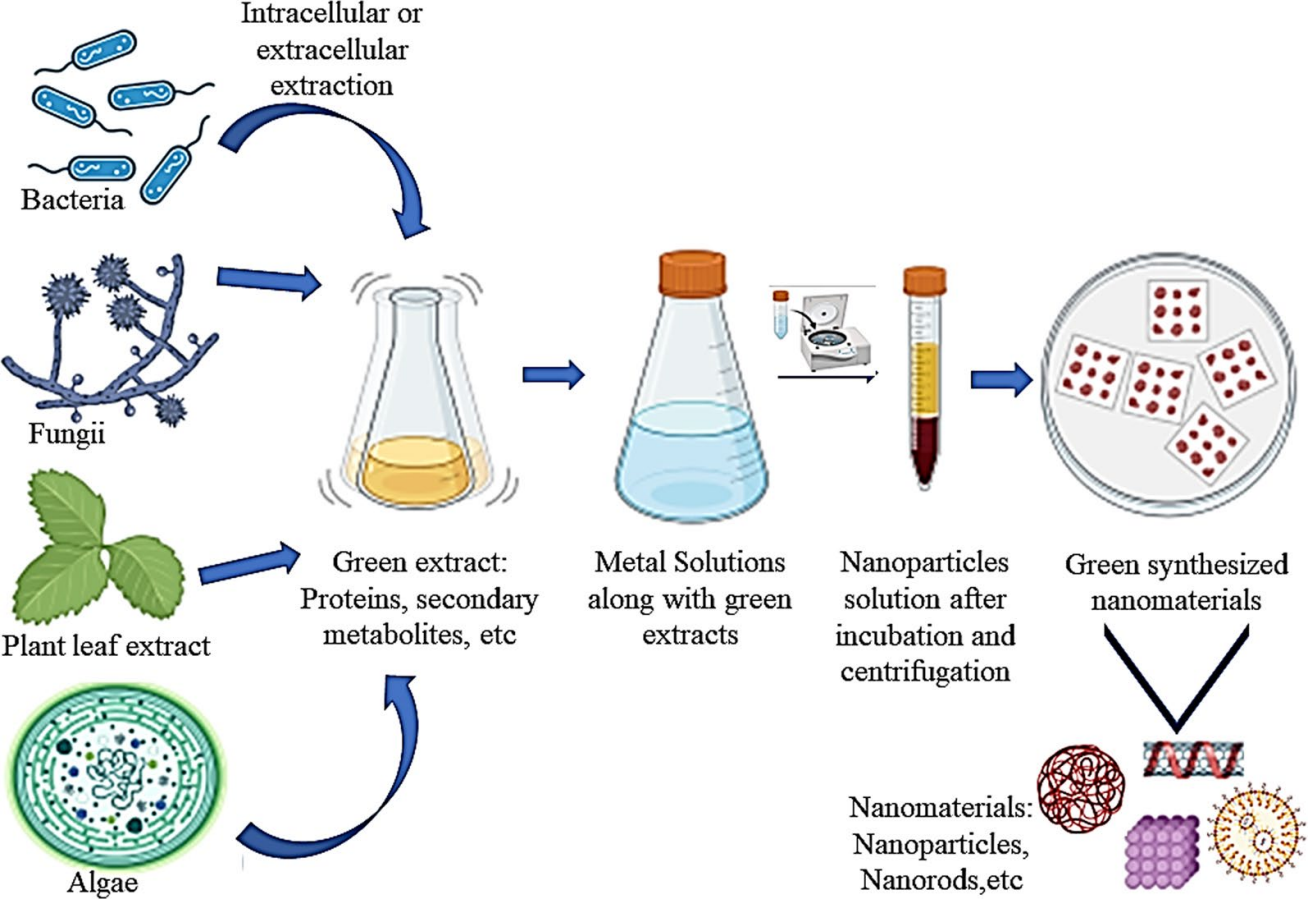
A generalized methods of green synthesis of nanomaterials via exploring biogenic agents

Functionalized green nanomaterials are nanoscale substances engineered using environmentally benign methods and materials. These nanomaterials are often synthesized using plant extracts, microorganisms, or other biological entities, ensuring that the production process is sustainable and minimizes ecological impact. The functionalization process involves the surface modification of these nanomaterials to enhance their stability, bioavailability, and interaction with plant tissues and pathogens. Functionalization further enhances the capabilities of green nanomaterials by attaching specific molecules to their surface, scientists can tailor their properties for specific applications. For example, functionalized nanomaterials can be designed to directly target and kill plant pathogens. Initially, NPs can be loaded with antimicrobial agents, antibiotics, and delivered directly to the site of infection [10], secondly, NPs trigger plant defence mechanisms. Certain nanomaterials can stimulate the plant’s immune system, enabling it to resist pathogen attacks more effectively [11] and thirdly, NPs act as carriers for nutrients or genetic materials; nanocarriers can deliver essential nutrients or protective genes directly to plant cells, enhancing their resilience against diseases [11]. Compared to physical and chemical methods of NPs synthesis, the green method uses less energy because it is a one-step, or “one pot” process. Green NPs have a very wide range of uses, from environmental sciences to medicine. A significant part of the green NP synthesis is played by the reducing and capping agents. Plants include substances that reduce the substrate to nanoscale and also cap it for stability. Figure 2 represent schematic representation of a generalized methos for biological synthesis of nanomaterials using different biological systems.

Plant-mediated synthesis of NPs involves the use of plant extract or its biomass to reduce metal ions into NPs. The reducing agents present in the plant extract, such as enzymes, alkaloids, and phenolic compounds, are responsible for the NPs formation [12]. Whereas, in case of microbial synthesis, different microbial systems like bacteria, fungi, and algae are usually used for the synthesis of various NPs [13, 14]. These organisms can reduce metal ions and stabilize the NPs using their metabolic processes. Enzyme-mediated synthesis employ enzymes which are highly specific catalysts that can control the size, shape, and properties of NPs [15]. This method offers a high degree of control over the NPs synthesis process.

## Advantages of green nanomaterials in plant disease management

Green NPs offers various advantages over other NPs such as improved energy efficiency, less amount of waste and greenhouse gas emission, and reduced consumption of non-renewable raw materials are the main advantages of green nanotechnology. Green synthesized NPs offers a prospect for overcoming the adverse effects before they occur.

*Targeted delivery and controlled release* Functionalized green nanomaterials can be designed to deliver active agents directly to the site of infection, ensuring higher efficacy at lower doses compared to conventional pesticides. This targeted approach minimizes collateral damage to beneficial microorganisms and reduces the overall chemical load on the environment.

*Enhanced antimicrobial properties* Many green nanomaterials possess inherent antimicrobial properties. For instance, silver and zinc oxide nanoparticles have been shown to exhibit strong bactericidal and fungicidal effects. Functionalization further enhances these properties, making them potent agents against a broad spectrum of plant pathogens.

*Induction of plant defence mechanisms* Certain nanomaterials can trigger systemic acquired resistance (SAR) in plants, enhancing their innate ability to resist infections. For example, nanoparticles functionalized with salicylic acid or chitosan can activate defence pathways, providing long-term protection against multiple diseases.

*Environmental sustainability* The use of green nanomaterials aligns with the principles of sustainable agriculture. By reducing reliance on synthetic chemicals and promoting natural plant defences, these nanomaterials help mitigate the adverse environmental impacts associated with conventional disease management practices.

## The potential of nanotechnology for plant disease control

**Fig. 3:**
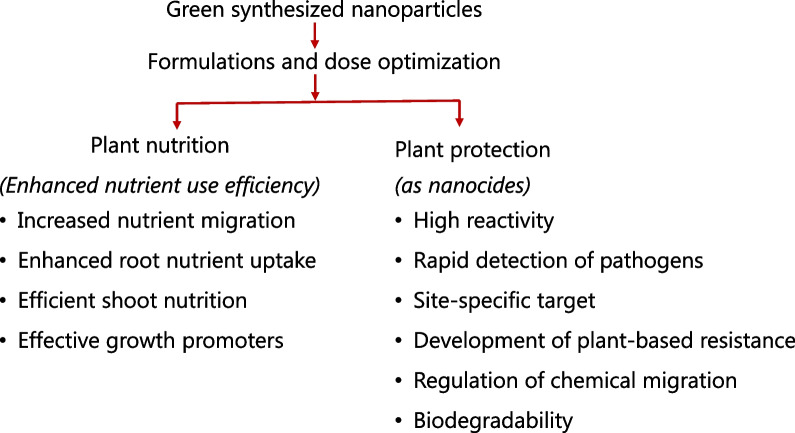
Applications of nanomaterials for plant disease management

The extraordinary reactivity and affectability of an NPs is a result of their minuscule size and substantial surface area which allow them to be employed against various biotic stresses with optimal and productive outcomes. The plants, soil, and hydroponic systems that suffer significant losses from a variety of phytopathogens, NPs possess the ability to eradicate specific microorganisms. In addition, nanomaterials have potential to reduce the requirement of agrochemicals which are commonly used in farming. There is a broad range of uses for metal oxide NPs carbon nanotubes, fullerenes, quantum dots, etc. The NPs play the role of nanocides thereby protecting the plants from various diseases through NPs site-specific and high reactivity, ability of NPs for development of plant-based resistance via mechanism of dsRNA, regulation of chemical migration and easily biodegradability of NPs. Apart from this, nanomaterials also enhance nutrient uptake of plants through root or foliar applications [16]. These both approaches i.e. plant protection and efficient nutrition ultimately result in enhanced crop yield (Fig. 3). When directly applied as soil amendments, foliar sprays, or seed primers, NPs have the potential to inhibit diseases in a similar manner that of chemical pesticides. The use of NPs in disease management involves three distinct mechanisms which involves i. the transport of active substances including micronutrients, insecticides, and elicitors, ii. bio-stimulants that promote plant innate immunity; and iii. antimicrobial potential [17]. Green NPs have shown to be extremely successful in fungicide/bactericide residue analysis as well as the diagnosis of plant diseases and infections. NPs protect crops, act as carriers for fungicide / bactericide or dsRNA, and can be delivered by foliar spraying, soaking, or drenching onto seeds or roots [18].

## Synthesis of metal NPs and its application for disease control

### Silver nanoparticles (AgNPs)

AgNPs have emerged as a promising tool in plant protection due to their unique antibacterial, antifungal, and antiviral properties. Here’s how AgNPs can be used in plant protection:

i. *Direct destruction* AgNPs disrupt the function of microbial cell membranes, leading to cell death. They can also release silver ions, further enhancing their antimicrobial activity.
ii. *Interference with metabolic processes* AgNPs can inhibit respiration, protein synthesis, and DNA replication in pathogenic organisms.
iii. *Stimulation of plant defence mechanisms* AgNPs can induce plant cells to produce defence compounds, strengthening their resistance to pathogens.

Green synthesis of AgNPs is simple one step, environment friendly method which makes NPs using different biological agents. It is reported that various metabolites such as proteins, enzymes, amino acids, polysaccharides, alkaloids, tannins, phenolics, saponins, terpenoids, and vitamins, paly important role in the synthesis of AgNPs [19]. According to Roy et al. [20] (alkaloids, flavonoids, and saponins present in *Eugenia jambolana* leaf extract are responsible for the synthesis of AgNPs. Because of their abundance in phytochemicals and secondary metabolites, plants offer a more effective and practical method for reducing and capping AgNPs. The selection of plant material for green AgNPs synthesis starts with identifying suitable plant species and parts. Characterization of the synthesized AgNPs can be achieved using various techniques such as UV spectroscopy, FTIS, TEM, X-ray diffraction, and SEM. According to Paosen et al. [21], these AgNPs syntheses have antibacterial action against *Enterococcus faecalis*, *Escherichia coli*, *Pseudomonas aeruginosa*, and *Klebsiella pneumonia*.

Highly stable AgNPs were produced by autoclaving a mixture of AgNO_3_, NaOH, and Laminaria extract with addition of alginic acid as a capping-reducing agent [22]. Laminaria extract contained relatively high levels of sapogenins, steroids, carbohydrates, and flavonoids acting as reducing agents and phytoconstituents acting as capping agents for AgNPs. The plant extract from *Boerhaavia diffusa* was used as a reducing agent for the green synthesis of AgNPs, which has antibacterial activity [23]. Green AgNPs offer healthier workplaces and communities, are affordable, energy-efficient, and cost-effective, and safeguard both the environment and human health. According to Ahmed et al. (2016), plants have an edge over other biological entities that preserve their culture and may lose their ability to synthesize NPs. An atomic force microscope and a UV–visible spectrophotometer were used to characterize the synthesized AgNPs [24, 25]. AgNPs were produced by Ponarulselvum et al. [26] using periwinkle (*Catheranthus roseus*) leaf extract and 1 mM silver nitrate; Banerjee et al. [27] used a similar procedure but used leaf extracts from *Azadirachta indica*, *Musa balbisiana*, and *Ocimum tenuiflorum*. An aqueous leaf extract of *Urtica dioica*, or common nettle, was used to create AgNPs [28]. There have been attempts to synthesize AgNPs from soybean seed extract, mango, neem leaves, and yellow oleander seeds too. Plant extract (10%) was employed as a reducing and capping agent, and silver nitrate (1 mM) as a metal source were used in the synthesis of AgNPs. For 1.0 min to 1.00 h, the reaction mixture was exposed to direct sunshine. The synthesis of AgNPs is significantly influenced by the intensity of light [29].

### Zinc-based NPs

Zinc-based NPs are emerging as exciting prospects for plant protection due to their unique properties and potential for sustainable pest and disease control. Here’s a closer look at their potential applications and mechanisms:

*Disease control* ZnO NPs exhibit antimicrobial, antifungal, and antiviral properties. They can be used to control fungal diseases like powdery mildew and anthracnose, as well as bacterial infections [30, 31]. Green-synthesized ZnO-NPs showed a greater antifungal efficacy than the chemically synthesized one against *A. citri*, the causal agent of citrus black rot [32].

*Nematode control* ZnO NPs have been shown to effectively control plant-parasitic nematodes, a major threat to agricultural productivity. They can also enhance plant defences against biotic and abiotic stresses [33].

Bacteria are among the greatest options for the synthesis of NPs because of their remarkable capacity to reduce heavy metal ions. The capacity of *B. subtilis*, *E. coli*, *B. cereus*, and *P. aeruginosa* to extract Ag^+^, Cu^2+^, Cd^2+^, and La3 + from solution was investigated by Mullen et al. [34]. It was discovered that significant amounts of metallic cations might be bound by bacterial cells. Furthermore, a subset of these bacteria (*P. fluorescens, B. subtilis, Trichoderma* spp., and *Saccharomyces* spp.), known as magnetotactic bacteria, are capable of producing inorganic materials such as internal magnetite NPs [35]. Using the extracellular culture filtrate of *Pichia fermentans* JA2 cultivated as culture suspension in yeast peptone glucose medium for 24 h, Chauhan et al. [36] manufactured the ZnO NPs.

### Copper nanoparticles

Nargund et al. [37] demonstrated the green synthesis of CuNPs using the aqueous extract of *Syzygium aromaticum* flower buds (clove) as a reducing agent and synthesized Cu NPs were found to have size less than 100 nm [37]. CuNPs synthesis using *Eucalyptus globule* leaf extract as a reducing agent and copper sulphate as a precursor. *Gloriosa superba L.* was used in the manufacture of copper oxide CuONPs, which had a size range of 30–70 nm and a round to slightly irregular form [38]. The *Gloriosa superba* L. extract was employed in smaller quantities (0.1 g/ml) to create CuO NPs ant it has shown antimicrobial activities against *Klebsiella aerogenes, Pseudomonas desmolyticum, and E. coli* [39].

CuNPs possess various mechanisms of action that contribute to their efficacy in plant protection, it mainly Direct Microbial Interaction. The direct microbial interaction usually happened through following different factors-

(i) *Membrane disruption* Cu NPs’ high surface area allows them to interact with microbial membranes, leading to leakage of vital cellular components and ultimately cell death [40].
(ii) *Reactive oxygen species (ROS) generation* Cu NPs interact with oxygen and organic matter, promoting the generation of ROS like hydroxyl radicals. These ROS damage microbial cell membranes, DNA, and proteins, causing oxidative stress and cell death [41]. Ameh et al. [42] reported Cu-NPs induced elevated levels of reactive oxygen species and antibacterial activities than the surface-stabilized silver nanoparticles.
(iii) *Enzyme inhibition* Cu NPs can bind to and inhibit essential enzymes in microbial metabolism, disrupting key cellular processes and preventing pathogen growth.
  *Nutrient competition* Cu NPs can adsorb essential nutrients like iron and phosphorus, making them unavailable to pathogens and hindering their growth and reproduction.
(iv) Plant Defense System Stimulation:
  *Induction of systemic acquired resistance (SAR)* Cu NPs can trigger the plant’s immune system to produce defense compounds like phytoalexins and pathogenesis-related proteins, enhancing its resistance to a wider range of pathogens [43].
(v) *Increased antioxidant activity* Cu NPs can stimulate the production of antioxidant enzymes in plant cells, scavenging ROS generated by pathogens and protecting plant tissues from oxidative damage. The study reported green synthesis Cu–O NPs showing high antibacterial, and antioxidant potency and less toxicity against *Bacillus cereus* and *Staphylococcus aureus* [44].
(vi) *Induction of cell wall reinforcement* Cu NPs can promote the accumulation of lignin and other cell wall components, creating a physical barrier against pathogen penetration [45].

### Chitosan and its derivatives nanoparticles

Chitosan, a naturally occurring biopolymer derived from chitin found in crustacean shells, possesses potent plant protection properties [46]. In recent years, research has focused on harnessing these properties through the development of chitosan-based nanoparticles (ChNPs). This exciting branch offers multiple mechanisms of action against various plant threats, making it a promising alternative to traditional pesticides.

**Fig. 4:**
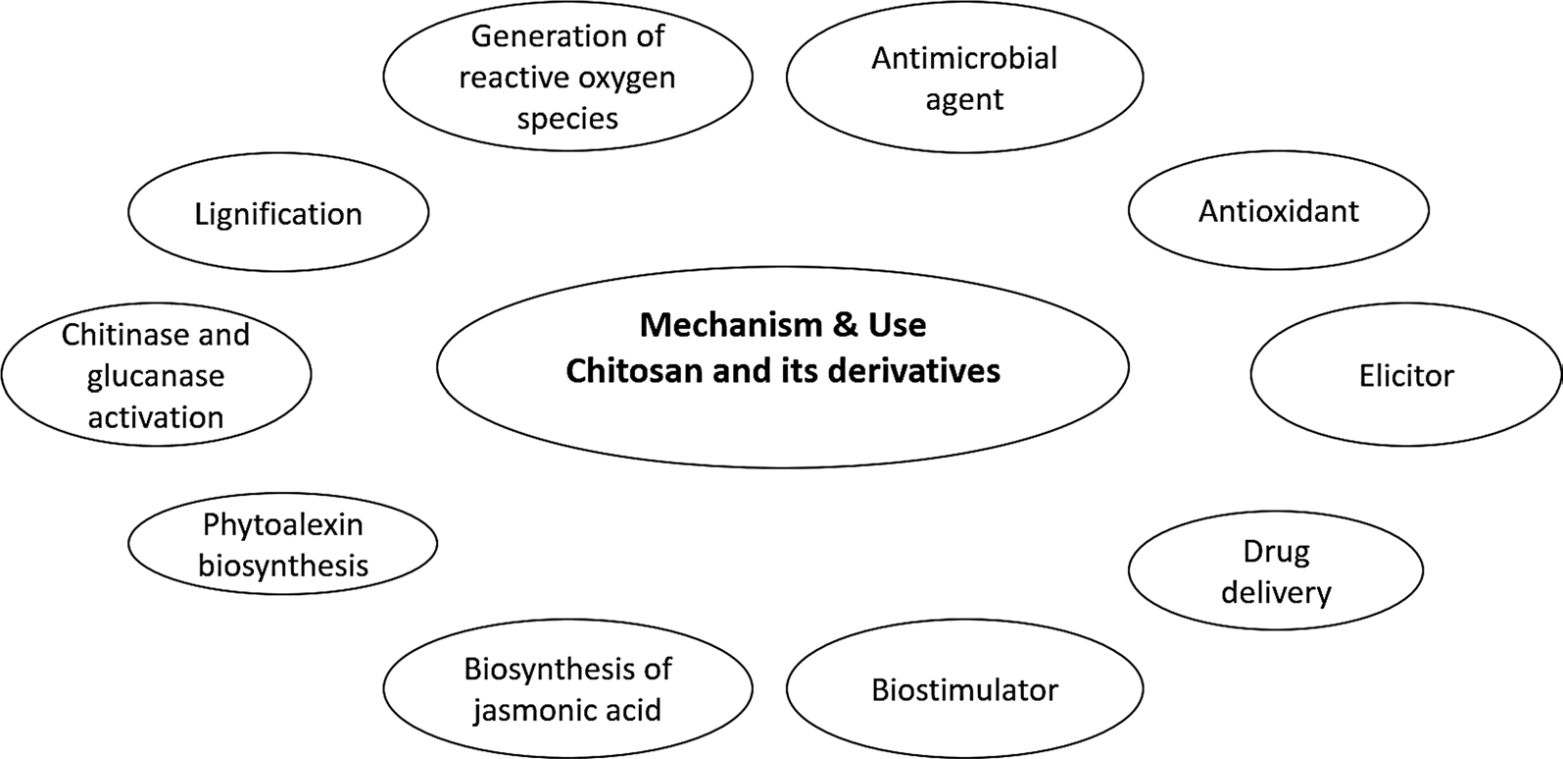
Chitosan as potent biomolecule for addressing the biotic and abiotic stresses in plants: mechanism and its applications

Because ChNPs are poorly soluble in aqueous solutions, they are typically combined with an organic or inorganic copolymer to increase their solubility. Nanomedicine, biomedical engineering, and the creation of novel therapeutic drug release systems with enhanced bioavailability, specificity, sensitivity, and decreased pharmacological toxicity have all shown a great deal of interest in ChNPs [47]. NPs used as soil additives to increase soil fertility in order to boost plant growth, abiotic stress tolerance, and seed germination [48]. Chitosan sticks to the leaf and stem epidermis, making it easier for the bioactive chemicals to be absorbed. In contrast, they are not as widely used in agriculture, particularly when it comes to managing plant diseases and insect pests. In *M. phaseolina*, *R. Solani*, and *A. alternata*, cu-chitosan NPs exhibit growth inhibition [49]. Based on the ionotropic gelation of chitosan and sodium tripolyphosphate, ChNPs were created [50]. As per Hassan and Chang [51], chitosan is the most prevalent naturally occurring polymer that hinders germination and causes disruptions to cell growth, sporulation, spore viability, and the induction of various defense responses in host plants. It also inhibits or induces different biochemical activities during the plant-pathogen interaction. In response to microbial infections, chitosan’s elicitor actions cause host plants to mount a range of defense mechanisms, such as the build-up of phytoalexins and lignin production, pathogen-related (PR) proteins and proteinase inhibitors, and the formation of callose [52]. Chitosan has antifungal and antibacterial properties and functions as a resistance elicitor, triggering both systemic and localized plant defensive responses. When used as a seed treatment, it has demonstrated efficacy against seed-borne infections and has been tested for the management of various pre- and post-harvest illnesses affecting a wide range of crops [53]. Under both in vitro and in vivo settings, chitosan is utilized to treat fenugreek seeds in order to manage the root rot disease caused by *Fusarium solani* [54]. The efficient binding of chitosan with RNA and its ability to permeate cell membranes have been made possible by the target-specific suppression of insect pests through the use of chitosan NP-entrapped siRNA delivery vehicles [55]. Numerous host defense mechanisms, including lignification, chitinase and glucanase activation, phytoalexin synthesis, reactive oxygen species production, and jasmonic acid synthesis, are stimulated by chitosan (Fig. 4).

#### ChNPs mechanisms of action

*Direct antimicrobial activity* ChNPs disrupt the cell membranes of pathogens like bacteria, fungi, and viruses, leading to leakage of cell contents and cell death [56, 57]. ChNPs chelate essential metal ions needed by pathogens for vital functions, starving them and inhibiting their growth. ChNPs generate ROS upon interaction with light or certain enzymes, causing oxidative stress and damaging pathogen cells [58].

*Stimulation of plant defense system* ChNPs trigger the plant’s defense system to produce defense compounds like phytoalexins and pathogenesis-related proteins, enhancing resistance to a broader range of pathogens. ChNPs activate signaling pathways in plant cells, leading to the production of protective enzymes and cell wall reinforcement, creating a physical barrier against pathogen invasion [59].

Physical barrier formation:

ChNPs form a thin film on plant surfaces, acting as a physical barrier against pathogen attachment and penetration. ChNPs can be formulated with specific polymers or biochar to enhance their adherence and provide prolonged protection [60].

Nutrient Delivery and Growth Promotion: ChNPs can act as a slow-release source of essential nutrients like nitrogen and phosphorus for plants, improving their overall health and resistance to stress. ChNPs can stimulate root growth and nutrient uptake, enhancing plant vigor and yield [48].

### Sulphur nanoparticles (SNPs)

Sulphur atoms combine to form sulphur nanoparticles (SNPs), which are particles at the nanoscale dimensions. The exceptional characteristics are due to their very small dimensions, extensive surface area, and quantum effects. SNPs can be developed through different techniques, such as chemical reduction, hydrothermal processes, and biological pathways. Recently, there has been a surge in the research towards SNPs and their utilization in the various fields like food, agriculture, and biomedical fields. This remarkable interest can be attributed due to the excellent antibacterial and anticancer properties exhibited by these nanomaterials [61]. One such notable example is development of nano-sulphur, it displays potential in combating plant pathogens and provides numerous advantages in promoting sustainable agriculture [62]. Understanding the mechanisms behind SNPs synthesis and their behaviour is crucial for harnessing their potential across various domains.

SNPs were produced by Awwad et al. [63] using sodium thiosulfate and fruit extract containing *Albizia julibriss*. Salem et al. [64] used citric acid, sodium thiosulfate pentahydrate (Na_2_S_2_O_3_·5H_2_O), and *Melia azedarach* leaf aqueous extract to generate SNPs. They used a mild inspiration method to allow the sulfur precipitations. SNPs were formed from sodium thiosulfate in the presence of pomegranate (*Punica granatum*) peel aqueous extract by Salem et al. [64]. Green SNPs were produced from neem and eucalyptus leaf extracts by Nargund et al. [25]. The reaction mixture’s hue changed to indicate the production of NPs. By using particle size analysis (PSA) and atomic force microscopy (AFM), accurate size and distribution were confirmed. Using PSA, the average diameter of SNPs was found to be 56.30 nm. Plant diseases are managed using fungicides depending on SNPs [65]. Choudhury et al. [66] demonstrated that nano-sulphur exhibits a stronger bactericidal impact on the facultative fungal food pathogen *Aspergillus niger* when compared to elemental sulfur. In a study conducted on tomato plants infected with *Fusarium oxysporum f. *sp.* lycopersici*, the foliar application and seed treatment with nano-sulfur resulted in a decrease in the occurrence of diseases. This reduction was attributed to a decrease in the accumulation of ROS in the plants and an increase in the levels of salicylic acid (SA), a plant hormone. In general, the breakdown of microbial cell membranes caused by SNPs can result in hindering the growth and reproduction of microorganisms by ultimately causing cell death [67]. In a study the in vitro antifungal assessment of ZnONPs and SNPs against phytopathogenic fungi of ginger, including *Aspergillus*, *Fusarium*, and *Pythium*, indicates their potential for developing alternative fungicidal materials for agricultural use. Statistical analysis shows that longer contact time between fungal species and ZnNPs or SNPs resulted in significant inhibition of fungal growth. This research suggests that these materials could be scaled up for commercial application in pre-treating seed ginger before planting [68]. In another study scientists have analyzed microbiome-pathogen-host interactions in the rhizosphere and recorded their reaction to the addition of sulphur in its bulk form, as well as sulphur nanoparticles coated with either pristine or stearic acid. It has been found that Nano-sulfur formulations induce distinct changes in the rhizosphere community when compared to conventional bulk sulphur, especially in relation to a plant pathogen, and offer promising prospects for the eco-friendly application of nanotechnology in agriculture [62].

## Nanomaterials and microorganism’s interaction: mechanism of action

**Fig. 5:**
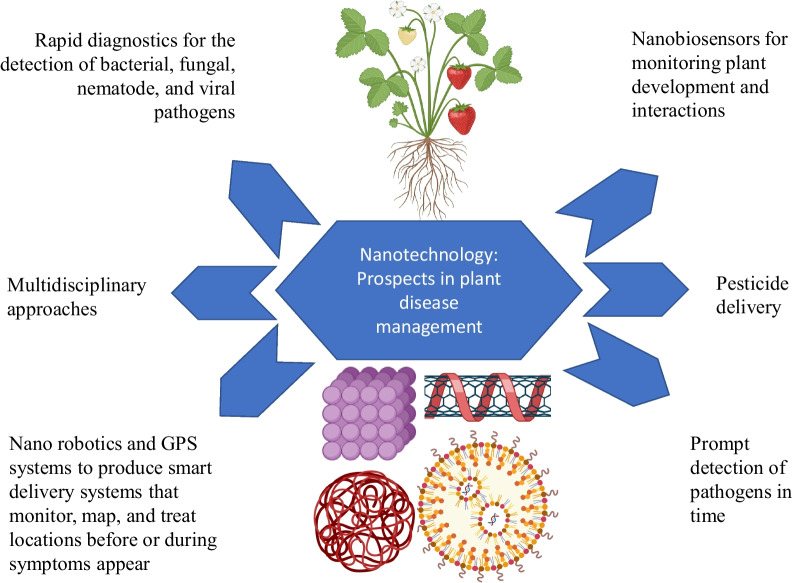
Prospects of nanotechnological aspects towards plant disease management

The interaction and mechanism underlying between nanomaterials and pathogens holds a greater area of importance in plant pathology research. Plant-based and Microorganism-derived NPs are regarded as a valuable resource for the synthesis of nanomaterials because they can be produced in large quantities and exhibit great bio-sorption ability. Many bacteria, including *E. coli, Lactobacillus casei, B. cereus, P. proteolytica, B. amyloliquefaciens, B. indicus, B. cloacae, Enterobacter, Geobacter* spp., and *Arthrobacter gangotriensis,* are used in the production of bio-reduced silver NPs [69]. It is possible to carry out the NP synthesis both intracellularly and extracellularly. The centrifuged culture filtrate and an aqueous metallic salt solution are combined for extracellular production. NP synthesis is indicated by the hue shift [70]. After growing microorganisms under ideal growth circumstances and incubating it with a metal ion solution, the biomass is thoroughly cleansed with sterile water for intracellular synthesis. The colour shift indicates the synthesis of nitrogen pollutants. After that, NPs are gathered using centrifugation, washing, and ultrasonication [71]. Due to their ability to synthesize bigger numbers of NPs than bacteria and the existence of a variety of intracellular enzymes, fungi are better biological agents for the creation of metal and metal oxide NPs. Nanoparticles possess the capability to directly engage with microbial cell membranes. Due to their nano dimensions, they can effortlessly infiltrate the cell wall and access the lipid bilayer. Once within, nanoparticles have the potential to impair the cell membrane’s integrity by either physically harming it or modifying its fluidity. Consequently, this interference hampers the cell’s capacity to uphold osmotic balance and facilitate nutrient transportation. Metal nanoparticles such as silver, copper, and zinc can release metal ions into their surrounding environment. These ions have the potential to interfere with microbial metabolic pathways by either disrupting enzyme activity or binding to essential biomolecules. As an example, silver ions can hinder bacterial respiration by attaching to cytochrome proteins within the electron transport chain. Certain nanoparticles, like silver nanoparticles (AgNPs), could produce ROS upon exposure to light or various environmental stimuli. ROS, which consist of superoxide radicals and hydrogen peroxide, are extremely reactive and have the potential to harm cellular biomolecules and structures such as proteins, lipids, and DNA. When microbes are subjected to ROS, they undergo oxidative stress, resulting in either cell death or stunted growth. Majorly all nanoparticles possess some surface charge, because of their nano dimensions and greater surface area. Therefore, when nanoparticles are positively charged, they could bind to the negatively charged surfaces of microbial cells. This binding has an impact on the viability and functionality of the cells. In addition, when nanoparticles aggregate, they can obstruct microbial attachment sites and nutrient uptake channels. Microorganisms can uptake nanoparticles either through endocytosis or passive diffusion. Upon entry, these nanoparticles have the potential to accumulate organelles such as mitochondria or lysosomes, as well as various cytoplasmic regions. This internal localization within the microorganism can lead to disturbances in organelle function, changes in pH levels, and ultimately impact microbial growth. Nanoparticles could interact with microbial DNA, resulting in DNA damage or mutations. Genotoxic effects may result in cell cycle arrest, apoptosis, or impaired replication. The level of genotoxicity is influenced by the type of nanoparticle, its concentration, and the duration of exposure. The prospects of nanotechnological aspect for plant disease management is highlighted in Fig. 5.

Nanoparticles possess a significant surface area-to-volume ratio, enabling them to efficiently cross cell membranes. Upon entry, they interfere with microbial cellular processes, block enzymes, and alter gene expression patterns. Most importantly, they can selectively adsorb metal ions and function well under a variety of environmental conditions, including temperature, pH, and ionic strength. They are also economically advantageous. The mechanism of action of nanoparticles on microorganisms is dependent on nanoparticle characteristics (such as size, shape, composition), the type of microbial species, and the surrounding environmental conditions. Researchers are actively undergoing investigations of these processes with an objective to gain a deeper insight into the interactions between nanoparticles and microbes, as well as their potential effects on both human health and ecosystems. The general mechanism of nanoparticles that accounts for their antimicrobial properties remains consistent with what was previously mentioned, however, their effects may vary when dealing with different types of microorganisms such as bacteria, fungi, and viruses.

### Impact on bacteria

Bacterial diseases have caused extensive harm to crops due to the development of antibacterial resistance, posing a significant risk to food availability. Consequently, several NPs with antibacterial properties have been developed to address the issue of plant diseases and antibiotic resistance while simultaneously enhancing crop productivity and bolstering plant immunity. NPs produced through green synthesis techniques have demonstrated superior efficacy and non-toxicity compared to those synthesized using chemical methods. These NPs, which are produced through environmentally friendly methods, have shown remarkable efficacy in terms of their bactericidal effect. They have the potential to revolutionize sustainable agriculture by increasing crop productivity and strengthening plant resistance [72].

**Fig. 6:**
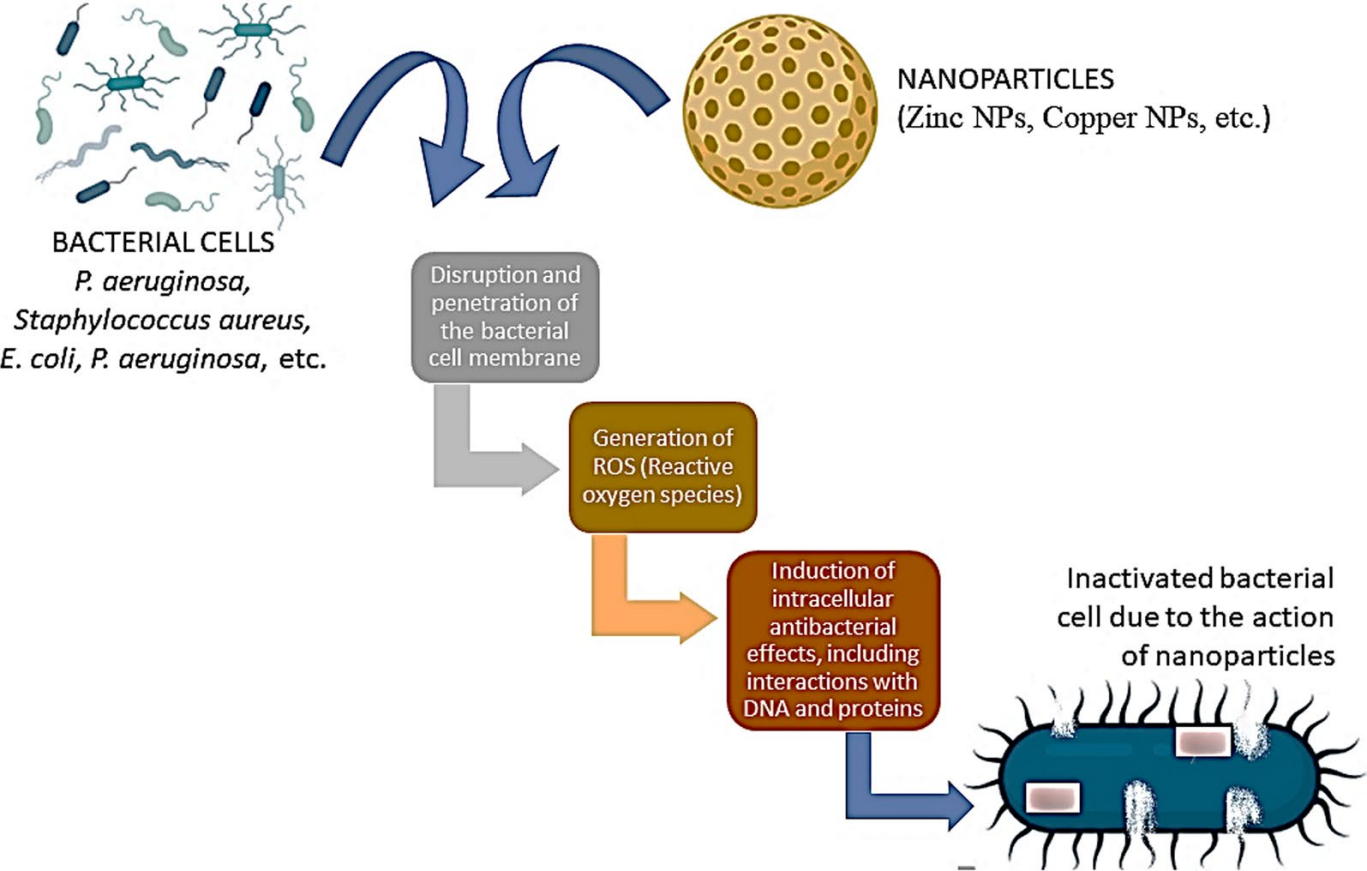
Schematic illustration of nanoparticle-mediated protection against bacteria

Green-synthesized nanoparticles (NPs) offer a more reliable and safer bactericidal effect against different bacteria that cause diseases in plants. NPs’ antibacterial action is deviated in a few ways, including the following:

1. Disruption and penetration of the bacterial cell membrane
2. ROS (Reactive Oxygen Species) production
3. Alteration of bacterial intracellular biomolecules (DNA and Protein) [73] (Fig. 6).
4. Modulating energy transduction pathway
5. Damage to the bacterial cell wall and disruption of peptidoglycan

Nanoparticles which are composed of various metals, including silver, magnesium, silicon, zinc, and carbon, have well demonstrated inhibitory effects on economically significant plant bacteria like *Xanthomonas citri pv. citri and Xylella fastidious* [8, 72]*.* Studies have shown that zinc NPs (NPs) work well against *B. subtilis* spores as well as *P. aeruginosa, S. aureus, E. coli* [74]*.* At all doses, the ZnO NPs demonstrated antibacterial activity against *Xanthomonas axonopodis pv. citri*. The minimum inhibitory concentrations of NPs against plant bacterial pathogens shown that nano-ZnO at concentrations ranging from 12.5 to 50 μg/ml inhibited every tested bacterium. Verticillium wilt and fusarium wilt have been studied in relation to *Xanthomonas perforans*. Zn NPs eliminate bacteria and fungi that cause rose leaf stains. According to reports, CuO NPs have antibacterial properties and can also combat *S. aureus, B. subtilis, P. aeruginosa, and E. coli* [75]. Studies have shown that CuNPs have a biocidal effect on bacteria phytopathogens like *Ralstonia solanacearum, Pseudomonas spp., and Xanthomonas spp* [76, 77]. The antibacterial action against Xac was investigated using green generated Cu NPs [37]. When compared to the antibiotic streptocycline, Cu NPs showed little antibacterial action against *R. solanacearum* and *X. campestris pv. citri*. But the study found that freshly made CuNPs for *Exserohilum turcicum* slowed down spore germination [25]. Chen et al. [78] synthesized copper oxide nanoparticles (CuONPs) with papaya leaf extracts and assessed their antibacterial effects on *Ralstonia solanacearum*, the pathogen responsible for bacterial wilt. They found that CuONPs proved potent antibacterial properties, leading to the complete eradication of all *R. solanacearum* strains following exposure to 250 μg/ml CuONPs. CuONPs were found to hinder biofilm formation, decrease swarming motility, and disrupt ATP production in bacterial cells. Transmission electron microscopy (TEM) analysis revealed severe nanomechanical damage to the cytomembrane of bacterial cells upon interaction with CuONPs, along with the absorption of numerous nanoparticles. Furthermore, they carried molecular investigations and found the downregulation of key pathogenesis and motility-related genes as part of the mechanism underlying CuONPs’ antimicrobial activity [78]. Silver nanoparticles are commonly utilized as antimicrobial agents to combat different plant pathogens. Ag Nps demonstrate remarkable bactericidal properties and are effective in managing diseases triggered by bacteria like *Pseudomonas syringae, Ralstonia solanacearum, and Xanthomonas axonopodis* [79]*.*

### Impact on fungi

Green functionalised nanoparticles have been extensively investigated for their capability in controlling fungal plant pathogens. They offer improved alternate of using chemical fungicides for controlling fungal plant diseases, leading to enhanced crop productivity and overall health. There are different mechanisms behind NPs antifungal effects on infective fungi such as Hypha plasmolysis, damage to fungal cell walls that results in cell death, disruption of plasma membrane permeability preventing proper functioning due to NPs protein attachment, DNA damage, disruption of the electron transport chain and protein oxidation, and the generation of ROS that cause cellular damage and impede nutrient uptake are among the common effects of metallic NPs [80]. Numerous fungi have been extensively studied and are involved in the biosynthesis of NPs. These include *F. oxysporum, Collitotrichum *sp.*, Trichothecium *sp.*, T. asperellum, T. viride, Phaenerochaete chryosporium, F. solani, F. semitectum, A. fumigatus, Coriolus versicolor, A. niger, Phoma glomerata, Penicillium brevicompactum, Cladosporium cladosporioidis, P. fellutanum,* and *Volvariella volvacea.* The unique properties of silver nanoparticles (AgNPs) have led to their recognition in the agricultural sector for combating fungal pathogens. Ag is the first NP applied to powdery mildew plant diseases [81]. AgNPs have exhibited the ability to hinder the growth of different types of fungi, such as *Aspergillus fumigatus, A. niger, A. flavus, Trichophyton rubrum, Candida albicans, and Penicillium species*. Their mechanism of action involves the generation of reactive oxygen species and free radicals, which subsequently result in protein denaturation, nucleic acid impairment, lipid peroxidation, and disruption of the cell wall. Ultimately, these processes lead to a modification in cell membrane permeability and ultimately culminate in cell death [82]. The fungicidal activity of Ag NPs/PVP was evaluated against a variety of yeasts and molds, including *Candida albicans, C. glabrata, C. krusei, C. tropicalis*, and *A. brasiliensis*. AgNPs were reported to decrease the activity of all tested pathogens by Krishnaraj et al. [83], investigated the impact of AgNPs on plant pathogenic fungi, including *Macrophomina phaseolina, Alternaria alternata, B. cinereal, Sclerotinia sclerotiorum, Rhizoctonia solani, and Curvularia lunata*. The antifungal activity of AgNPs against *Colletotrichum gloesporioides*, a fungal strain from papaya fruits exhibiting anthracnose symptoms, was assessed by Miguel et al. [84]. AgNPs were assessed in vitro and in vivo by Lamsal et al. [85] in relation to powdery mildew in cucumber and pumpkin grown under various conditions. AgNPs demonstrated the greatest resistance to fungal hyphae growth and conidial germination in vitro, as well as the highest disease inhibition rate in the field. AgNPs were assessed in vitro against *X. campestris pv. malvacearum (Xcm)* by Rajesh et al. [86]. Without AgNPs, Xcm entered an exponential phase very quickly. Nevertheless, exposure to AgNPs stopped Xcm from growing. In order to assess the antifungal properties of AgNPs provided by Bio-Plus Co. Ltd. (Pohang, Korea) against pepper anthracnose, Lamsal et al. [87] applied different concentrations of the material. When compared to the control in vitro, AgNPs (100 ppm) produced the greatest inhibition of fungal hyphae development and conidial germination. In field tests, using the treatment prior to plant disease outbreaks resulted in noticeably greater fungal suppression. According to SEM data, AgNPs had a negative impact on Colletotrichum spp. mycelium growth. PDA was significantly inhibited by 100 ppm AgNPs in vitro experiments. Under field conditions, plants treated with 50 ppm AgNPs prior to the disease outbreak showed the lowest disease incidence (9.7%). According to Poovizhi and Krishnaveni [88], ZnONPs exhibit antifungal action against *Aspergillus* sp. and *Penicillium* sp. by significantly inhibiting spore germination and mycelial inhibition. Wagner et al. (2016) assessed the effectiveness of Zn and ZnONPs against *Peronospora tabacina*, an oomycete pathogen that causes tobacco blue mold. ZnONPs are utilized to control citrus greening, while gold NPs and Fusarium wilt of watermelon are employed to identify viral infections in plants. ZnONPs caused cellular dysfunctions that resulted in fungal hyphae distortion, which impeded the growth. *A. flavus* colonization was inhibited by the ZnONPs (25 mg/ml) [89]. ZnONPs have been shown to be effective against two pathogenic fungus species, *F. oxysporum* and *P. expansum*, at a concentration of 12 mg/l, according to Yehia and Ahmed’s [89] research. In sunflowers, Mn and Zn also inhibited the pathogens that cause damping off and charcoal rot [90]. According to Giannousi et al. [91], Cu NPs are very successful at controlling plant diseases caused by *Phytophthora infestans*. Field pea rust disease was shown to be most effectively controlled by CuSO_4_ and Na_2_B_4_O_7_ [92]. ChNPs were investigated by Saharan et al. [49] for their antifungal effects against *Macrophomina phaseolina, A. alternata*, and *R. solani* at different doses ranging from 0.001 to 0.1 percent. Copper nanoparticles with different sizes (11–14 nm) and chemical compositions (Cu_2_O, CuO, and Cu/Cu_2_O) have been documented to effectively manage *Phytophthora* infestans. These nanoparticles require lower amounts (15–35 g/hl) compared to the approved copper-based products (35–224 g/hl) [91]. The in vitro antifungal activity of synthesized SNPs and three other commercial products, namely commercial Nano-Sulphur, commercial Sulphur, and Sulphur 80 WP, against powdery mildew of okra (*Erysiphe cichoracearum*) was assessed at 1000 ppm. When compared to the control, sulfur fungicides dramatically decreased the germination of *E. cichoracearum* conidia. Conidial germination was lowest in laboratory-synthesised nano-sulfur (4.56%), with Canadian nano-sulfur (14.17%), Merck sulfur (15.53%), sulfur 80 WP (15.97%), and control (23.09%) following. In addition to preventing conidial germination, interaction with nanosulfur also caused disruptions to cleistothecial appendages, resulting in sterile cleistothecia. According to the study, lab-synthesized nanosulphur outperformed commercial formulations in terms of effectiveness [93]. Plant disease control employs NPs of Al, Mg, Fe, Si, Mn, and other elements. Si has drawn attention to safeguard plant health because of its important role in plant defense. *Xanthomonas, Aspergillus, Botrytis cinerea, and Fusarium spp*. are restricted by carbon NPs [94]. The green synthesis of CuONPs with phoma extract showed notable antifungal effectiveness against *Aspergillus flavus* and *Aspergillus niger.* Through the serial dilution technique, they found, the minimum inhibitory concentration (MIC) be 180 µg/ml for *A. niger* and 220 µg/ml for *A. flavus*. MIC values showed a significant difference when compared to the standard fungicide mancozeb [95].

### Impact on viruses

Nanoparticles have the potential to act as inhibitory agents against a variety of microorganisms, such as bacteria, algae, archaea, fungi, and a broad spectrum of viruses. There is limited chance of managing plant viruses indirectly by regulating their insect vectors, such as aphids, thrips, mites, whiteflies, etc., as the drugs needed to treat plant virus infections are not readily available. Nevertheless, despite their many benefits such as their wide availability, quick action, and dependability organic and inorganic pesticides have detrimental side effects on creatures that are not their intended targets. The inhibition of viral infection process by nanoparticles comprises of various mechanisms [96].

New ideas that are created with advantageous features, such as adjustable pore size, shape, and surface properties, have been made possible by nanotechnology. These ideas can be employed as carriers of active ingredients, such as viricides, pesticides, and double-stranded RNA (dsRNA), for precise and targeted delivery through adsorption, encapsulation, and conjugation, or as protectants against insect vectors, viruses, and virus diseases. The potential for dsRNA delivery by nanotechnology in environmental settings and the activation of RNA interference (RNAi) in plants to provide viral infection resistance [97]. NPs have the ability to interact with host plants, viruses, and their vectors. The metallic NPs investigate the antiviral function of NPs in plants that obstruct viral replication through various methods, as well as NP-plant-virus interactions. Phytoviruses have been the subject of little research. A potential management strategy for combating plant viral illnesses is “nanophytovirology,” which entails practices for the identification, diagnosis, and control of plant viral diseases and associated pathogens in order to prevent epidemic infections [98]. The outermost layer of viruses is made up primarily of capsid proteins, which have a highly reactive surface that can mix with metallic ions and serve as attachment sites for components that are dispersed in nanoscale. Nanowires and nanotubes are synthesized using viruses as a template. Graphene-based silver nanocomposites have the ability to suppress tomato bushy stunt virus (TBSV) in lettuce by reducing the virus concentration and disease severity. Both the foliar treatment and the soil replenishment of the NPs were proven to be effective. When *Cucumis sativus* was treated with SiO_2_ NPs by soil supplementation, the symptoms of the Papaya ringspot virus (PRSV) infection were considerably reduced. According to Elbeshehy et al. [99], faba bean plants that were simultaneously sprayed with AgNPs and inoculated with bean yellow mosaic virus showed noticeably better outcomes when the NPs were applied 24 h after the infection, as opposed to when they were sprayed before or at the same time as the infection. The tomato spot wilt virus (TSWV) was significantly inhibited from *Chenopodium amaranticolor* plants. Within 24 h of the tomato bushy stunt virus inoculation, potato plants sprayed with AgNPs showed a decrease in both the virus concentration and the illness percentage. The BBTV genotoxicity caused by banana plants treated with 50 ppm AgNPs after BBTV inoculation was reduced by using AgNPs as a novel, safe, and effective antiviral drug [99]. It has also been observed that tomato plants sprayed with SiO_2_NPs and cucumber plants treated with NiONPs have less severe cases of the tomato yellow leaf curl virus. TMV was unable to propagate to primordial leaves thanks to the ZnO and SiO_2_NPs. Turnip mosaic virus (TuMV) antiviral activity was observed in tobacco pre-treated with Fe_2_O_3_ or TiO_2_NPs, indicating that the NPs disrupted the virus’s defensive mechanisms and post-translational modification or protein manufacturing processes. When AgNPs were sprayed on tomatoes infected with PVY and ToMV, the NPs bonded to the virus and hindered its reproduction, reducing the severity of the disease and its viral burden. The sun-hemp rosette virus was totally repressed and the signs of the bean yellow mosaic virus were postponed when the silver nanoparticle spray was applied to bean leaves. The application of mechanical abrasion to introduce gold NPs conferred resistance against the Barley yellow mosaic virus. ChNPs protect alfalfa, peanuts, potatoes, and cucumbers from Alfalfa mosaic virus infection, thereby inducing viral resistance in plants. When cluster bean leaves were sprayed with a 50 ppm aqueous solution of silver NPs after being inoculated with the sun hemp rosette virus (SHRV), the disease was completely suppressed [100]. When BioClay NPs containing dsRNA were sprayed on the challenged plants, they demonstrated resistance to the viruses that cause pepper mild mottle virus (PMMoV) and cucumber mosaic virus (CMV) over a period of 20 days, or almost three weeks, in contrast to the control group [99]. In the study conducted by Alkubaisi and Aref [101], it was demonstrated that AuNPs have the ability to induce damage to the virus-like particles (VLPs) of the barley yellow dwarf virus-PAV. Through the use of TEM, the researchers observed the presence of puffed and deteriorated VLPs adorned with AuNPs, as well as completely destroyed and vanished particles. AgNPs have the ability to attach to the coat protein virus particles of the tomato mosaic virus (ToMV) and potato virus Y (PVY) [102]. Cai et al. [103] studied the effect of Fe_3_O_4_ NPs on *Nicotiana benthamiana* plants through foliar applications. They found that Fe_3_O_4_ NPs induce the generation of ROS, thereby enhancing the functioning of antioxidant enzymes and triggering SA-dependent signaling pathways. Prolonged exposure to Fe_3_O_4_ NPs stimulates plant growth and stimulates the defense response against plant viruses like TMV (*Tobacco mosaic virus*) [104]. Recently Zhu et al. [105] developed a novel sodium alginate nanogel composite (ALGNP), incorporating Zn2 + NP and ε-PL(ε-poly-l-lysine). They found that Zn2 + @ALGNP@PL exhibited enhanced antiviral efficacy in comparison to an equal quantity of Zn2 + and the commercial antiviral drug LNT (Table 1).

**Table 1:** Green nanomaterials for successfully applied for disease management in various plants

SN	Nanoparticle type	Targeted pathogens	Mode of action	Reported outcomes	References
1	AgNPs	*Alternaria solani*	Production of phytochemicals phenols and flavonoids shows antifungal properties	Significant reduction in disease severity index (DSI) (73%) and disease incidence (DI) (69%) compared to the control plants	[106]
2	AgNPs	*Penicillium *sp.*, Aspergillus *sp.*, Fusarium *sp.*,* and* Ralstonia solanacearum*	Production of phytochemicals phenols and flavonoids and secondary metabolites shows antifungal properties	Reduced the growth of *Penicillium *sp. (92%), *Fusarium* sp. (89%), *Aspergillus* sp. (69%)	[107]
3	AgNPs	*Rhizoctonia solani, Fusarium oxysporum, Sclerotinia sclerotiorum,* and* Sclerotium rolfsii*	Production of phytochemicals phenols and flavonoids and secondary metabolites shows antifungal properties	AgNPs at 100 ppm showed significantly higher efficacy in inhibiting mycelial growth of the pathogens as compared to the Carbendazim at 3000 ppm	[108]
4	AgNPs	*Colletotrichum* species	Detrimental effect on mycelial growth	AgNPs at 100 ppm concentration produced maximum inhibition of the growth of fungal hyphae as well as conidial germination	[87]
5	AgNPs	Tomato mosaic virus (ToMV) and Potato virus Y (PVY)	Systemic acquired resistance (SAR) AgNPs interact with the polypeptide coats of tomato mosaic virus (ToMV) and potato virus Y, damage to TMV shell proteins, agglomeration, and even disintegration	Disease severity as well as relative concentration of both viruses were decreased dramatically by the treatment of AgNPs at 50 ppm	[102]
6	AgNPs	Sunhemp rosette virus	AgNPs bind to the virus body and inactivates the virus by inhibiting virus replication in host plant	Spray AgNPs 50 ppm showed complete suppression of the disease	[109]
7	AgNPs	Tomato spotted wilt virus (TSWV)	Inhibition of local lesions and reduction in TSWV infection	The higher inhibitory effect (90.4%) was recorded with sprayed AgNPs	[110]
8	AgNPs	Potato virus Y (PVY)	Induction of resistance in host plant against virus	AgNPs at 0.1 µg/µl concentration led to decrease in virus concentration and infection percentage	[111]
9	ZnO NPs	*Aspergillus flavus* and* Aspergillus niger*	Antifungal activity	Highest zone of inhibition was observed in 25 μg/ml of 27 ± 5 nm size ZnONPs against *Aspergillus flavus* and* Aspergillus niger*	[112]
10	ZnO NPs	*Pseudomonas aeruginosa* and* Aspergillus flavus*	Antibacterial and Antifungal activity	The maximum zone of inhibition was observed in the ZnO NPs (25 μg/ml) against *Pseudomonas aeruginosa* (22 ± 1.8 mm) and *Aspergillus flavus* (19 ± 1.0 mm)	[89]
11	ZnO NPs	*Xanthomonas oryzae pv. oryzae*	Reactive oxygen species based on their antibacterial efficiency and the deformity in the cell wall	ZnO NPs inhibition effects against Xoo strain GZ 0006 at a concentration of 16.0 μg/ml and the biofilm formation was decreased by 74.5%	[113]
12	Fe_3_O_4_NPs	Tobacco mosaic virus	Reactive oxygen species accumulation, peroxidase activity, catalase activity and systemic resistance-related genes (PR1 and PR2)	Fe_3_O_4_NPs foliar spraying 12 days markedly inhibited TMV replication	[103]
13	CuNPs	*Macrophomina phaseolina, Bipolaris maydis, Fusarium verticillioides, Rhizoctonia solani* and* Erwinia carotovora*	Antimicrobial inhibition	CuNPs exhibited significant inhibition at 20 ppm against *Macrophomina phaseolina, Bipolaris maydis,* and *Fusarium verticillioides*, and at 50 ppm against Rhizoctonia solani and shows bactericidal property against *Erwinia carotovora* and *Ralstonia solanacearum* at 30 ppm	[114]
14	SNPs	*Fusarium oxysporum f. *sp.* lycopersici*	Activated the salicylic acid-dependent systemic acquired resistance pathway and upregulation of the expression of pathogenesis-related and antioxidase-related genes	1 mg/plant of 30 nm SNPs (30-SNPs) exhibited decreasing disease incidence by 47.6%	[67]
15	SNPs	*Botrytis cinerea* and* Sclerotinia sclerotiorum*	Upregulation of total phenolic (TPC) and total soluble solids (TSS) Content components	S-NPs exhibited the highest antifungal activity	[115]
16	Cu-NPs	*Botrytis cinerea* and* Sclerotinia sclerotiorum*	Upregulation of total phenolic (TPC) and total soluble solids (TSS) content components	Cu-NPs exhibited the antifungal activity	[115]
17	Ch-CuNPs	*Pyricularia grisea*	Significant increase in defense enzymes	Application of Ch-CuNPs nearly 75% protection was observed in finger millet plants	[116]
18	Ch-CuNPs	*Rhizoctonia solani* and* Pythium aphanidermatum*	Mycelium showed cellular content leakage	98% mycelial growth inhibition at 0.1% Ch-CuNPs	[117]

## Comparative analysis of nanoparticles for plant protection

Nanoparticles have emerged as a promising tool in plant protection due to their unique properties and potential for targeted action. Here’s a comparison of some commonly explored nanoparticles for their effectiveness in this field:

Nanoparticles:

- *Silver nanoparticles (AgNPs)* Well-known for their broad-spectrum antimicrobial activity, AgNPs can combat various plant pathogens like bacteria, fungi, and viruses. AgNPs—disrupt microbial membranes, However, concerns exist regarding their potential environmental toxicity [118].
- *Zinc- and Iron-based Nanoparticles (ZnNPs & FeNPs)* These nanoparticles offer a dual benefit. ZnNPs act as micronutrients essential for plant growth and can also exhibit antifungal properties. ZnNPs—stimulate plant defense systems. FeNPs can improve plant defense mechanisms and nutrient uptake. Both require careful control to avoid unintended phytotoxicity (plant toxicity) [119].
- *Copper nanoparticles (CuNPs)* Similar to AgNPs, CuNPs possess strong antimicrobial properties against bacteria and fungi. However, CuNPs can also be phytotoxic at high concentrations [114].
- *Chitosan nanoparticles* Derived from chitin (a natural biopolymer), chitosan nanoparticles offer a biodegradable and eco-friendly approach. They can act as carriers for other pesticides or enhance plant defense mechanisms [120].
- *Sulfur nanoparticles* Effective against specific fungal groups. They can release sulfur directly to the target site, potentially improving efficacy compared to traditional sulfur-based fungicides [62, 121].
- *Gold nanoparticles* Gold nanoparticles have the potential to serve as carriers for the active components of pesticides or chemicals that stimulate defense mechanisms in plant [122]. Gold nanoparticles (AuNPs) are being increasingly recognized as a viable solution for the control of plant diseases, owing to their distinctive characteristics. AuNPs possess inherent antibacterial properties that directly combat plant pathogens, effectively impeding the growth and propagation of both bacteria and fungi. By disrupting cell membranes and interfering with crucial cellular mechanisms, these nanoparticles effectively suppress the activity of pathogens, thereby mitigating their harmful effects. In addition to their direct antibacterial action, AuNPs also contribute to the enhancement of plant growth and bolstering stress resistance. Notably, researchers have observed significant improvements in seed germination, root development, and overall plant health when AuNPs are present. This underscores the potential of AuNPs as a valuable tool in promoting plant vitality and combating plant diseases [123].
- *Silica nanoparticles* Silica nanoparticles (SiO_2_ NPs) are utilized as effective carriers for delivering agro-products in pest management [16]. SiO_2_ NPs have emerged as an innovative and eco-friendly method to combat plant diseases. By applying SiO_2_ NPs through foliar treatment, the severity of rice blast fungus (*Magnaporthe oryzae*) disease can be significantly reduced by approximately 70% within a suitable concentration range. SiO_2_ NPs exhibit great potential as a sustainable remedy to improve plant health and strengthen their resilience [124].
- *Carbon nanotubes (CNTs)* CNTs, which are cylindrical formations composed of rolled graphene sheets, possess nanoscale diameters ranging from 1 to 100 nm have demonstrated potential in the suppression of plant diseases and the improvement of plant health [125]. Due to their diminutive size, they are capable of effectively infiltrating plant tissues and reaching the specific location of infection. Moreover, the extensive surface area of CNTs offers numerous contact points for interactions between plant cells and pathogens. CNTs enhance the activation of defense-related genes and trigger the production of ROS, thereby stimulating plant defense mechanisms [126].
- *Graphene nanoparticles* Graphene nanoparticles (GNPs) demonstrate impressive antibacterial and antifungal properties when combating plant pathogens. GNPs are safe and non-toxic for the environment. Additionally, GNPs have the ability to encase antimicrobial substances, thereby boosting their efficacy against pathogens [127]. GNPs possess distinct characteristics that render them promising contenders for the treatment of various diseases [128].

Nanoparticles provide various opportunities for sustainable and efficient plant disease control. Utilizing them can result in decreased environmental harm and improved management of pesticide-resistant plant pathogens (Table 2).

**Table 2:** Comparative analysis of nanoparticles for plant disease management

Property	AgNPs	ZnNPs & FeNPs	CuNPs	Chitosan NPs	Sulfur NPs
Antimicrobial Activity	Broad-spectrum (bacteria, fungi, viruses)	Moderate (fungi)	Broad-spectrum (bacteria, fungi)	Low-moderate	Fungus-specific
Plant Nutrition	None	Micronutrient source (Zn)	None	None	None
Biodegradability	Low	Moderate	Low	High	Moderate
Environmental Impact	Concerns regarding toxicity	May require controlled release	Concerns regarding phytotoxicity	Eco-friendly	Potential for soil acidification

## Nanofungicides nano-fungicides with enhanced efficiency over conventional fungicides

Hydrophobic fungicides can benefit from microencapsulation, which enhances their aqueous phase dispersion and permits a careful release of the active ingredient. Consistent release of the active component into the root zone will increase the fungicides effectiveness, lower the amount needed, and ultimately lessen its negative impacts on the environment [129, 130]. Nanofungicides are an innovative field of study within agriculture, utilizing nanotechnology to improve disease management. By employing nanomaterials as carriers for the active substances, nanotechnology can achieve this regulated release of the active ingredient. Lower dosages of pesticides, herbicides, and fertilizers can be safely and effectively delivered with the help of nanomaterials, which also reduce toxicity. Their droplet sizes are consistent and incredibly small, and their viscosity is reduced and their stability is higher. As a better pesticide delivery system, nanofungicides cover large areas, require fewer applications, increase protection for longer periods of time, have low viscosity, high kinetic stability, optical transparency, and boost the efficacy of fungicides due to their microbial, plant- or animal-based, eco-friendly nature [131]. In addition to exhibiting beneficial qualities such biodegradability, permeability, stiffness, thermal stability, solubility, and crystallinity, nanofungicides formulations can improve the solubility, dispersion, and wettability of agricultural formulations [132]. Effective techniques of delivering nanofungicides for plant protection include nanoemulsions, nanoencapsulated nanocages, and nanocontainers [132, 133]. Nanoemulsions consist of stable emulsions, either oil-in-water or water-in-oil, with droplets that are in the nanometer range. These emulsions can encapsulate active ingredients, thereby enhancing their solubility and bioavailability. Nanosuspensions comprise of solid nanoparticles that are suspended in a liquid carrier. These formulations enhance the stability and controlled release of active ingredients. By enabling targeted delivery and sustained action against pathogens, nanosuspensions offer significant advantages. Nanofungicides are designed with controlled release mechanisms to slowly release active compounds, providing extended protection and minimizing the necessity for frequent reapplications. Instances of this sustained release method involve nanoparticles incorporated into polymer matrices or enveloped in biodegradable substances [134]. Fungicide active ingredients can be mixed directly with nanoparticles in solid-based formulations. Nanomaterials such as aluminum oxide, zinc oxide, titanium dioxide, and silver have been extensively researched for their effectiveness against plant pathogens. By incorporating these nanoparticles, stability is enhanced, dosage requirements are reduced, and disease management is improved [135].

Nanotechnology holds enormous revolutionary promise for use in the food, medical, and agricultural sectors. Applications of nanotechnology in crop protection include the use of nanosensors for early detection of plant diseases and contaminants, such as pesticide residues, and encapsulated pesticides, fertilizers, and other agrochemicals for past disease management [136]. According to Mousavi and Rezaei [137], nanomaterials are increasing the fungicidal potency and delivery. When tested against *F. oxysporum* and *A. parasiticus*, Kumar et al. [138] found that when carbendazim was loaded with polymeric NPs, the fungal inhibition rate was higher than when carbendazim was used alone. In another study Bhattacharyya et al. [139] conducted a study on the effectiveness of copper oxide NPs against the fungal pathogen *F. oxysporum* causing *Fusarium wilt* in tomato plants. Their research revealed that the copper oxide NPs displayed potent antifungal properties by successfully managing the disease with a notably superior efficacy when compared to traditional copper-based fungicides[139]. Recently, in research it has been successfully developed the advancement of a pH-responsive fungicide nanoformulation using hollow mesoporous silica nanoparticles (HMSNs) as a nanocarrier [140]. Thereafter, the prochloraz (Pro) and ZnO quantum dots (ZnO QDs) were loaded over resulting nanocarrier. Their findings demonstrated that the prepared nanopesticide exhibits a high loading efficiency (24.96%) for Pro. In comparison to Pro, the deterioration rate of Pro loaded in nanocarrier after 24 h of ultraviolet exposure was lessen by 26.4%, evidently representing improved photostability through nanocarriers. Further, the release of nanofungicide under weak acidic environment after 48 h was 2.67 times higher than that in a neutral condition. This study highlighted the excellent pH-responsive and photostability characteristic of nanofungicides over native fungicide [140].

## Green nanotechnology: limitations and future directions

1. Nanomaterials may potentially harm a beneficial soil organism, the earthworm and insects.
2. There are increased safety concerns regarding nanomaterials in food and agriculture due to the most common exposure routes and factors involved in nanotoxicity.
3. Engineered nanomaterials are entering the environment due to the overuse of modern technologies.
4. Green NPs utilizes plant extract/biological agents as reducing or capping agent, but the constituents of bioagents are beyond control therefore the reproducibility and efficacies have limitations.
5. Green NPs scalability and cost currently limit the application of nanocarriers in agriculture.
6. Commercialization of nanomaterials for agricultural applications demands well-protected materials, superior testing significance, a clear-cut risk evaluation, and international supervisory regulation.

## Potential challenges and limitations of nanotech in disease management

While nanotechnology offers exciting possibilities in disease management, there are significant challenges to overcome before widespread adoption:

1. *Cost* Developing and producing NPs can be expensive due to specialized equipment, complex manufacturing processes, and the need for high purity materials. This can limit their affordability and accessibility, particularly in resource-limited settings.
2. *Scalability* Scaling up production from lab-scale synthesis to large-scale manufacturing can be difficult. Maintaining consistent quality and precise control over nanoparticle properties becomes a challenge at larger scales.
3. *Efficacy* Despite promising research, the therapeutic efficacy of some nanomedicines in humans needs further validation. Clinical trials are crucial to confirm their safety and effectiveness against specific diseases.
4. *Regulatory considerations* Regulatory frameworks for nanomedicines are still evolving. Defining safety standards, conducting thorough risk assessments, and establishing clear approval pathways are ongoing challenges.
5. *Potential environmental impacts* The long-term environmental impact of releasing NPs into the environment is not fully understood. Concerns exist regarding their potential to accumulate in the food chain or disrupt ecological processes. More research is needed to address these concerns.
6. *Unforeseen toxicity* Some NPs may exhibit unintended toxicity even at low doses. This can be due to factors like their size, shape, surface chemistry, and interaction with biological systems. Careful evaluation and safety testing are crucial before clinical use.

## Nanotechnology: limitations and future directions

7. Nanomaterials could potentially harm a beneficial soil organism, the earthworm.
8. There are increased safety concerns regarding nanomaterials in food and agriculture due to the most common exposure routes and factors involved in nanotoxicity.
9. Engineered nanomaterials are entering the environment due to the overuse of modern technologies.
10. Scalability and cost currently limit the application of nanocarriers in agriculture.
11. Commercialization of nanomaterials for agricultural applications demands well-protected materials, superior testing significance, a clear-cut risk evaluation, and international supervisory regulation.

## Future directions of nanotechnology in plant disease management

1. *Targeted delivery systems* Researchers are exploring surface modifications on NPs to recognize and bind to unique markers on the surface of bacteria, fungi, or viruses. This targeted approach could significantly reduce the dosage required and minimize potential harm to healthy plant tissues.
2. *Smart biocontrols* Encapsulation of beneficial bacteria or fungi within NPs could enhance their delivery, protection from harsh environmental conditions, and targeted release near the pathogen. This could lead to more effective and long-lasting biocontrol solutions.
3. *Plant-responsive nanomaterials* Researchers are exploring the development of NPs that can sense the presence of pathogens and trigger the plant’s own defense mechanisms. These “smart” NPs could act as sentinels, alerting the plant to potential threats and initiating a targeted defense response.

## Conclusion

Green nanotechnology is believed to address some of the present plant pathological issues by facilitating the early detection of plant diseases. It is one of the tools to be used to understand the host–pathogen interaction in a precise manner. It explains methods utilized for synthesis of various NPs, including: AgNPs, ZnONPs-and Fe_3_O_4_NPs, CuNPs, ChNPs, SNPs. Exploiting these nanomaterials to their full potential in the context of plant disease management. The positive impact of these NPs against bacteria, fungi, viruses and its use as fungicides and bactericide. Green synthesis is the most promising and environmentally friendly for the synthesis of NPs. Green nanotechnology applications in disease management play a significant role in increasing farm inputs and producing better-quality products. To conserve the novelty and quality of green nanotechnology, thorough research, and legislative development are needed in the future. Nanomaterials are expected to have an enormous impact on the rating and environmental affability of crop protection practices. The effect of NPs needs to be evaluated in terms biosafety assessment and regulations to confirm its effect on environment which will make this technology more proficient. Green synthesized NPs will be efficient and promising alternatives for present chemical formulations used in plant disease management.

## Data Availability

The datasets generated and analysed in this study are available from the corresponding author upon reasonable request.

## References

[1] Alexandratos N (1999). World food and agriculture: outlook for the medium and longer term. Proc Natl Acad Sci.

[2] Alexandratos N, Bruinsma J (2012). World agriculture towards 2030/2050: The 2012 Revision.

[3] Cardoza C, Nagtode V, Pratap A, Mali SN (2022). Emerging applications of nanotechnology in cosmeceutical health science: latest updates. Health Sci Rev.

[4] Sundararajan N, Habeebsheriff HS, Dhanabalan K, Cong VH, Wong LS, Rajamani R, Dhar BK (2024). Mitigating global challenges: harnessing green synthesized nanomaterials for sustainable crop production systems. Global Chall.

[5] Sarwar S, Raja DA, Hussain D, Shah MR, Malik MI. Introduction to Nanotechnology. In: Handbook of nanomaterials: electronics, information technology, energy, transportation, and consumer products, vol. 1; 2024. P. 1–26.

[6] Manjunatha SB, Biradar DP, Aladakatti YR, Manjunatha SB, Aladakatti YR (2016). Nanotechnology and Its Applications in Agriculture: A Review. J farm Sci..

[7] Sinha K, Ghosh J, Sil PC. New pesticides: a cutting-edge view of contributions from nanotechnology for the development of sustainable agricultural pest control. In: New pesticides and soil sensors; 2017. p. 47–79.

[8] Elmer W, White JC (2018). The future of nanotechnology in plant pathology. Annu Rev Phytopathol.

[9] Vijayaram S, Razafindralambo H, Sun YZ, Vasantharaj S, Ghafarifarsani H, Hoseinifar SH, Raeeszadeh M (2023). Applications of green synthesized metal nanoparticles—a review. Biol Trace Elem Res.

[10] Weldick PJ, Wang A, Halbus AF, Paunov VN (2022). Emerging nanotechnologies for targeting antimicrobial resistance. Nanoscale.

[11] Singh R, Choudhary P, Kumar S, Daima HK. Mechanistic approaches for crosstalk between nanomaterials and plants: plant immunomodulation, defense mechanisms, stress resilience, toxicity, and perspectives. Environ Sci Nano. 2024.

[12] Taha A, Farooq N, Hashmi AA. Medicinal plant-mediated nanomaterials. Green Synthesis of Nanomaterials; 2024. p. 22–45.

[13] Arteaga-Castrejón AA, Agarwal V, Khandual S (2024). Microalgae as a potential natural source for the green synthesis of nanoparticles. Chem Commun.

[14] Vikanksha, Kumar A, Singh J. Microbial synthesized nanoparticles in environment management. In: Microbiome-based decontamination of environmental pollutants; 2024. p. 381–401.

[15] Sanket S, Das SK. Role of enzymes in synthesis of nanoparticles. In: Bioprospecting of enzymes in industry, healthcare and sustainable environment; 2021. p. 139–153.

[16] Khan M, Khan AU, Parveen A. Nanoparticles in plant disease management. In: Plant and nanoparticles; 2022. p. 53–65.

[17] Garole VJ, Kondulkar S (2020). Phytoconstituents for nanoparticles synthesis: mini review. World J Pharm Res.

[18] Ahsan T, Yuanhua W (2021). Plant virus disease management by two modern applications (DsRNA nano-clay sheet and CRISPR/Cas). Arch Phytopathol Plant Prot.

[19] Zuhrotun A, Oktaviani DJ, Hasanah AN (2023). Biosynthesis of gold and silver nanoparticles using phytochemical compounds. Molecules.

[20] Roy PSD, Sharma N, Salaria KH, Chalotra R, Padekar SK, Guleria S (2021). Green synthesis of silver nano particles: recent advances and applications. Indian J Agric Biochem.

[21] Paosen S, Saising J, Wira Septama A, Piyawan VS (2017). Green synthesis of silver nanoparticles using plants from myrtaceae family and characterization of their antibacterial activity. Mater Lett.

[22] Kaidi S, Belattmania Z, Bentiss F, Jama C, Reani A, Sabour B. Synthesis and characterization of silver nanoparticles using alginate from the brown seaweed Laminaria Ochroleuca: structural features and antibacterial activity; 2021.

[23] Ahmed S, Ahmad M, Swami BL, Ikram S (2016). A review on plants extract mediated synthesis of silver nanoparticles for antimicrobial applications: a green expertise. J Adv Res.

[24] Swamy C, Nargund VB (2017). Sunlight induced mediated silver nanoparticles from seeds of *Thevetia peruviana* L., characterization and their antifungal efficacy against Curvularia Lunata (Wakker) Boedijn. Int J Curr Microbiol App Sci.

[25] Nargund V. Potential applications of green nanotechnology in crop protection; 2016.

[26] Ponarulselvam S, Panneerselvam C, Murugan K, Aarthi N, Kalimuthu K, Thangamani S (2012). Synthesis of silver nanoparticles using leaves of *Catharanthus roseus* Linn. G. Don and their antiplasmodial activities. Asian Pac J Trop Biomed.

[27] Banerjee P, Satapathy M, Mukhopahayay A, Das P (2014). Leaf extract mediated green synthesis of silver nanoparticles from widely available indian plants: synthesis, characterization, antimicrobial property and toxicity analysis. Bioresour Bioprocess.

[28] Jyoti K, Baunthiyal M, Singh A (2016). Characterization of silver nanoparticles synthesized using *Urtica dioica* Linn. leaves and their synergistic effects with antibiotics. J Radiat Res Appl Sci.

[29] Nargund V. Phytomining based nanotechnology: a novel approach in plant protection; 2016.

[30] Aydoğdu B, Aytar M, Ünal İ (2024). Comparison of characteristics and antimicrobial activity of synthesized zinc oxide and magnetite iron oxide nanoparticles using four different plant extracts. Cumhuriyet Sci J.

[31] Nandhini M, Rajini SB, Udayashankar AC, Niranjana SR, Lund OS, Shetty HS, Prakash HS (2019). Biofabricated zinc oxide nanoparticles as an eco-friendly alternative for growth promotion and management of downy mildew of pearl millet. Crop Prot.

[32] Elshafie HS, El-Saber MM, Camele I, Abbas E (2023). Antifungal activity of green and chemically synthesized ZnO Nanoparticles against *Alternaria citri*, the causal agent citrus black rot. Plant Pathol J.

[33] El-Ansary MSM, Hamouda RA, Elshamy MM (2022). Using biosynthesized zinc oxide nanoparticles as a pesticide to alleviate the toxicity on banana infested with parasitic-nematode. Waste Biomass Valoriz.

[34] Mullen MD, Wolf DC, Ferris FG, Beveridge TJ, Flemming CA, Bailey GW (1989). Bacterial sorption of heavy metals. Appl Environ Microbiol.

[35] Kirthi AV, Rahuman AA, Rajakumar G, Marimuthu S, Santhoshkumar T, Jayaseelan C, Velayutham K (2011). Acaricidal, pediculocidal and larvicidal activity of synthesized ZnO nanoparticles using wet chemical route against blood feeding parasites. Parasitol Res.

[36] Chauhan R, Reddy A, Abraham J (2015). Biosynthesis of silver and zinc oxide nanoparticles using pichia fermentans JA2 and their antimicrobial property. Appl Nanosci.

[37] Nargund VB, Chikkanna S, Hulagappa, Pradeep M. Green synthesis, characterization of copper nanoparticles using *Syzygium aromaticum* L. and their efficacy against *Exserohilum turcicum* (Pass.) Leonard and Suggs. In: International conference on nanomaterials and nanotechnology (NANO-2015) proceedings on bio-nanomaterials for biomedical technology, K.S.R. College of Technology, 7–10th December Tamil Nadu; 2015. p. 155–158.

[38] Chikkanna S, Nargund VB, Madhu SG, Hasansab AN, Hulagappa. Synthesis of soybean mediated silver nanoparticles and its application against bacterial blight of pomegranate. In: 8th Bangalore India Nano 2016, 3^rd^–5th March, Bangalore, POS.23; 2016.

[39] Naika HR, Lingaraju K, Manjunath K, Kumar D, Nagaraju G, Suresh D, Nagabhushana H (2015). Green synthesis of CuO nanoparticles using *Gloriosa superba* L. extract and their antibacterial activity. J Taibah Univ Sci.

[40] Thahira Khatoon U, Velidandi A, Nageswara Rao GVS (2024). Nano-sized copper particles: chemical synthesis, characterization, and their size and surface charge dependent antibacterial potential. Results Chem.

[41] Sajjad H, Sajjad A, Haya RT, Khan MM, Zia M (2023). Copper oxide nanoparticles: in vitro and in vivo toxicity, mechanisms of action and factors influencing their toxicology. Comp Biochem Physiol Part C Toxicol Pharmacol.

[42] Ameh T, Gibb M, Stevens D, Pradhan SH, Braswell E, Sayes CM. Silver and copper nanoparticles induce oxidative stress in bacteria and mammalian cells; 2022. 10.3390/nano12142402 PMC9319685 35889626

[43] Ingle PU, Shende SS, Shingote PR, Mishra SS, Sarda V, Wasule DL, Rajput VD, Minkina T, Rai M, Sushkova S, Mandzhieva S, Gade A (2017). Chitosan nanoparticles (ChNPs): a versatile growth promoter in modern agricultural production☆. Heliyon.

[44] Kaningini AG, Motlhalamme T, More GK, Mohale KC, Maaza M (2023). Antimicrobial, antioxidant, and cytotoxic properties of biosynthesized copper oxide nanoparticles (CuO-NPs) using athrixia phylicoides DC. Heliyon.

[45] Li K, Zhong W, Li P, Ren J, Jiang K, Wu W (2023). Antibacterial mechanism of lignin and lignin-based antimicrobial materials in different fields. Int J Biol Macromol.

[46] Boamah P, Onumah J, Aduguba W, Santo K (2023). Application of depolymerized chitosan in crop production: a review. Int J Biol Macromol.

[47] Sharifi-Rad J, Quispe C, Butnariu M, Rotariu LS, Sytar O, Sestito S, Rapposelli S, Akram M, Iqbal M, Krishna A, Kumar NVA, Braga SS, Cardoso SM, Jafernik K, Ekiert H, Cruz-Martins N, Szopa A, Villagran M, Mardones L, Martorell M, Docea AO, Calina D (2021). Chitosan nanoparticles as a promising tool in nanomedicine with particular emphasis on oncological treatment. Cancer Cell Int.

[48] Ingle PU, Shende SS, Shingote PR, Mishra SS, Sarda V, Wasule DL, Rajput VD, Minkina T, Rai M, Sushkova S, Mandzhieva S, Gade A (2022). Chitosan nanoparticles (ChNPs): a versatile growth promoter in modern agricultural production. Heliyon.

[49] Saharan V, Mehrotra A, Khatik R, Rawal P, Sharma SS, Pal A (2013). Synthesis of chitosan based nanoparticles and their in vitro evaluation against phytopathogenic fungi. Int J Biol Macromol.

[50] Du WL, Niu SS, Xu YL, Xu ZR, Fan CL (2009). Antibacterial activity of chitosan tripolyphosphate nanoparticles loaded with various metal ions. Carbohydr Polym.

[51] Hassan O, Chang T (2017). Chitosan for eco-friendly control of plant disease. Asian J Plant Pathol.

[52] El Hadrami A, Adam LR, El Hadrami I, Daayf F (2010). Chitosan in plant protection. Mar Drugs.

[53] Orzali L, Corsi B, Forni C, Riccioni L. Chitosan in agriculture: a new challenge for managing plant disease. In: Biological activities and application of marine polysaccharides; 2017.

[54] Ghule MR, Ramteke PK, Ramteke SD, Kodre PS, Langote A, Gaikwad AV, Holkar SK, Jambhekar H (2021). Impact of chitosan seed treatment of fenugreek for management of root rot disease caused by fusarium solani under in vitro and in vivo conditions. 3 Biotech.

[55] Zhang X, Zhang J, Zhu KY (2010). Chitosan/double-stranded RNA nanoparticle-mediated RNA interference to silence chitin synthase genes through larval feeding in the African malaria mosquito (*Anopheles gambiae*). Insect Mol Biol.

[56] Chatterjee A, Bhattacharya S, Sur R (2023). Alginate/chitosan/diallyl disulfide nanoparticles: synthesis, characterization and their anti-inflammatory efficacy on TPA-induced acute mouse ear inflammation. Carbohydr Polym Technol Appl.

[57] Xing Y, Wang X, Guo X, Yang P, Yu J, Shui Y, Chen C, Li X, Xu Q, Xu L, Bi X, Liu X (2021). Comparison of antimicrobial activity of chitosan nanoparticles against bacteria and fungi. Coatings.

[58] Sathiyabama M, Indhumathi M (2022). Chitosan thiamine nanoparticles intervene innate immunomodulation during chickpea-fusarium interaction. Int J Biol Macromol.

[59] Chun SC, Chandrasekaran M (2019). Chitosan and chitosan nanoparticles induced expression of pathogenesis-related proteins genes enhances biotic stress tolerance in tomato. Int J Biol Macromol.

[60] Correa-Pacheco ZN, Bautista-Baños S, de Lorena-Ramos-García M, del Carmen-Martínez-González M, Hernández-Romano J (2019). Physicochemical characterization and antimicrobial activity of edible propolis-chitosan nanoparticle films. Prog Org Coat.

[61] Shankar S, Jaiswal L, Rhim JW (2021). New insight into sulfur nanoparticles: synthesis and applications. Crit Rev Environ Sci Technol.

[62] Steven B, Hassani MA, LaReau JC, Wang Y, White JC (2024). Nanoscale sulfur alters the bacterial and eukaryotic communities of the tomato rhizosphere and their interactions with a fungal pathogen. NanoImpact.

[63] Awwad AM, Salem NM, Abdeen AO (2015). Noval approach for synthesis sulfur (S-NPs) nanoparticles using *Albizia julibrissin* fruits extract. Adv Mater Lett.

[64] Salem NM, Albanna LS, Awwad AM (2016). Green synthesis of sulfur nanoparticles using *Punica granatum* peels and the effects on the growth of tomato by foliar spray applications. Environ Nanotechnol Monit Manag.

[65] Rao KJ, Paria S (2013). Use of sulfur nanoparticles as a green pesticide on fusarium solani and venturia inaequalis phytopathogens. RSC Adv.

[66] Choudhury SR, Mandal A, Ghosh M, Basu S, Chakravorty D, Goswami A (2013). Investigation of antimicrobial physiology of orthorhombic and monoclinic nanoallotropes of sulfur at the interface of transcriptome and metabolome. Appl Microbiol Biotechnol.

[67] Cao X, Wang C, Luo X, Yue L, White JC, Elmer W, Dhankher OP, Wang Z, Xing B (2021). Elemental sulfur nanoparticles enhance disease resistance in tomatoes. ACS Nano.

[68] Ingle PU, Rai M, Golińska P, Gade AK (2024). Phytomediated zinc oxide and sulfur nanoparticles for management of soft-rot causing pathogenic fungi in ginger. Biocatal Agric Biotechnol.

[69] Satapathy P, Aishwarya S, Rashmi Shetty M, Akshaya Simha N, Dhanapal G, Aishwarya Shree R, Biswas A, Kounaina K, Patil AG, Avinash MG, Devi AT, Gopal S, Nagendra Prasad MN, Veena SM, Hudeda SP, Muthuchelian K, More SS, Melappa G, Zameer F (2020). Phyto-nano-antimicrobials: synthesis, characterization, discovery, and advances. Front Anti-Infect Drug Discov.

[70] Ibrahim E, Zhang M, Zhang Y, Hossain A, Qiu W, Chen Y, Wang Y, Wu W, Sun G, Li B (2020). Green-synthesization of silver nanoparticles using endophytic bacteria isolated from garlic and its antifungal activity against wheat fusarium head blight pathogen *Fusarium graminearum*. Nanomaterials.

[71] Soni M, Mehta P, Soni A, Goswami G (2019). green nanoparticles: synthesis and applications. IOSR J Biotechnol Biochem.

[72] Gul M, Khan RS, Islam ZU, Khan S, Shumaila A, Umar S, Khan S, Brekhna, Zahoor M, Ditta A (2024). nanoparticles in plant resistance against bacterial pathogens: current status and future prospects. Mol Biol Rep.

[73] Wang L, Hu C, Shao L (2017). The antimicrobial activity of nanoparticles: present situation and prospects for the future. Int J Nanomed.

[74] Bryaskova R, Pencheva D, Nikolov S, Kantardjiev T (2011). Synthesis and comparative study on the antimicrobial activity of hybrid materials based on silver nanoparticles (AgNps) stabilized by polyvinylpyrrolidone (PVP). J Chem Biol.

[75] Angrasan VMSR (2014). Biosynthesis of copper nanoparticles by vitis vinifera leaf aqueous extract and its antibacterial activity. Int J Curr Microbiol App Sci.

[76] Sundin GW, Castiblanco LF, Yuan X, Zeng Q, Yang CH (2016). Bacterial disease management: challenges, experience, innovation and future prospects. Mol Plant Pathol.

[77] Banik S, Pérez-de-luque A (2017). In vitro effects of copper nanoparticles on plant pathogens, beneficial microbes and crop plants. Span J Agric Res.

[78] Chen J, Mao S, Xu Z, Ding W (2019). Various antibacterial mechanisms of biosynthesized copper oxide nanoparticles against soilborne *Ralstonia solanacearum*. RSC Adv.

[79] Ul Abadin Z, Ahmad W, Ullah A, Tanveer NA, Hanif S, Amjad S. Exploring nanoparticles for effective management of plant pathogens: a comprehensive review, vol. 19. 2023.

[80] Das K, Kanti Jhan P, Chandra Das S, Aminuzzaman FM, Yaw Ayim B. Nanotechnology: past, present and future prospects in crop protection. Technology in Agriculture; 2021.

[81] Park HJ, Kim SH, Kim HJ, Choi SH (2006). A new composition of nanosized silica-silver for control of various plant diseases. Plant Pathol J.

[82] Mansoor S, Zahoor I, Baba TR, Padder SA, Bhat ZA, Koul AM, Jiang L (2021). Fabrication of silver nanoparticles against fungal pathogens. Front Nanotechnol.

[83] Krishnaraj C, Ramachandran R, Mohan K, Kalaichelvan PT (2012). Optimization for rapid synthesis of silver nanoparticles and its effect on phytopathogenic fungi. Spectrochim Acta A Mol Biomol Spectrosc.

[84] Aguilar-Méndez MA, Martín-Martínez ES, Ortega-Arroyo L, Cobián-Portillo G, Sánchez-Espíndola E (2011). Synthesis and characterization of silver nanoparticles: effect on phytopathogen colletotrichum gloesporioides. J Nanopart Res.

[85] Lamsal K, Kim SW, Jung JH, Kim YS, Kim KS, Lee YS (2011). Inhibition effects of silver nanoparticles against powdery mildews on cucumber and pumpkin. Mycobiology.

[86] Rajesh M (2016). Synthesis, characterization of copper nanoparticles. Int Adv Res J Sci Engg Tech.

[87] Lamsal K, Kim SW, Jung JH, Kim YS, Kim KS, Lee YS (2011). Application of silver nanoparticles for the control of colletotrichum species in vitro and pepper anthracnose disease in field. Mycobiology.

[88] Poovizhi J, Krishnaveni B (2015). Synthesis, characterization, and antimicrobial activity of zinc oxide nanoparticles synthesized from calotropis procera. Int J Pharm Sci Drug Res.

[89] Jayaseelan C, Rahuman AA, Kirthi AV, Marimuthu S, Santhoshkumar T, Bagavan A, Gaurav K, Karthik L, Rao KVB (2012). Novel microbial route to synthesize ZnO nanoparticles using aeromonas hydrophila and their activity against pathogenic bacteria and fungi. Spectrochim Acta A Mol Biomol Spectrosc.

[90] Abd El-Hai KM, El-Metwally MA, El-Baz SM, Zeid AM (2009). The use of antioxidants and microelements for controlling damping-off caused by rhizoctonia solani and charcoal rot caused by macrophomina phasoliana on sunflower. Plant Pathol J.

[91] Giannousi K, Avramidis I, Dendrinou-Samara C (2013). Synthesis, characterization and evaluation of copper based nanoparticles as agrochemicals against phytophthora infestans. RSC Adv.

[92] Singh D, Kumar A, Singh AK, Tripathi HS (2013). Induction of resistance in field pea against rust disease through various chemicals/ micronutrients and their impact on growth and yield. Plant Pathol J.

[93] Gogoi R, Singh P, Kumar R, Nair K, Alam MdI, Srivastava C, Yadav S, Gopal M, Roy Choudhury S, Goswami A (2013). Suitability of nano-sulphur for biorational management of powdery mildew of okra (*Abelmoschus esculentus* Moench) Caused by *Erysiphe cichoracearum*. Plant Pathol Microbiol.

[94] Elmer W, Ma C, White J (2018). Nanoparticles for plant disease management. Curr Opin Environ Sci Health.

[95] Ingle PU, Shende SS, Hande D, Rai M, Golinska P, Gade AK (2024). Mycogenic copper oxide nanoparticles for fungal infection management in agricultural crop plants. Bionanoscience.

[96] Hussain FS, Abro NQ, Ahmed N, Memon SQ, Memon N (2022). Nano-antivirals: a comprehensive review. Front Nanotechnol.

[97] Carvalho R, Jones JB, Paret ML. Utility of nanoparticles in management of plant viruses. In: Nanotechnology-based sustainable alternatives for the management of plant diseases; 2022. p. 233–241.

[98] Farooq T, Adeel M, He Z, Umar M, Shakoor N, Da Silva W, Elmer W, White JC, Rui Y (2021). Nanotechnology and plant viruses: an emerging disease management approach for resistant pathogens. ACS Nano.

[99] Elbeshehy EKF, Elazzazy AM, Aggelis G (2015). Silver nanoparticles synthesis mediated by new isolates of Bacillus spp. nanoparticle characterization and their activity against bean yellow mosaic virus and human pathogens. Front Microbiol.

[100] Jain D (2014). Green synthesis of silver nanoparticles and their application in plant virus inhibition. J Mycol Plant Pathol.

[101] Alkubaisi NA, Aref NMA (2017). Dispersed gold nanoparticles potentially ruin gold barley yellow dwarf virus and eliminate virus infectivity hazards. Appl Nanosci.

[102] Noha K, Bondok AM, El-Dougdoug KA (2018). Evaluation of silver nanoparticles as antiviral agent against ToMV and PVY in tomato plants. Sciences.

[103] Cai L, Liu C, Fan G, Liu C, Sun X (2019). Preventing viral disease by ZnONPs through directly deactivating TMV and activating plant immunity in nicotiana benthamiana. Environ Sci Nano.

[104] Cai L, Cai L, Jia H, Liu C, Wang D, Sun X (2020). Foliar exposure of Fe_3_O_4_ nanoparticles on *Nicotiana benthamiana*: evidence for nanoparticles uptake, plant growth promoter and defense response elicitor against plant virus. J Hazard Mater.

[105] Zhu X, He W, Wang J, Liu C, Pei Y, Wen Y, Wang X, Chen H, Wang H, Ran M, Ma X, Sun X (2024). A high rain-erosion resistant bio-based nanogel with continuous immunity induction for plant virus inhibition. Int J Biol Macromol.

[106] Ansari M, Ahmed S, Abbasi A, Hamad NA, Ali HM, Khan MT, Haq IU, Zaman QU (2023). Green synthesized silver nanoparticles: a novel approach for the enhanced growth and yield of tomato against early blight disease. Microorganisms.

[107] Haroon M, Zaidi A, Ahmed B, Rizvi A, Khan MS, Musarrat J (2019). Effective inhibition of phytopathogenic microbes by eco-friendly leaf extract mediated silver nanoparticles (AgNPs). Indian J Microbiol.

[108] Kaman PK, Dutta P (2019). Synthesis, characterization and antifungal activity of biosynthesized silver nanoparticle. Indian Phytopathol.

[109] Jain D, Kothari SL (2014). Green synthesis of silver nanoparticles and their application in plant virus inhibition. J Mycol Plant Pathol.

[110] Shafie RM, Salama AM, Farroh KY (2018). Silver nanoparticles activity against tomato spotted wilt virus. Middle East J Agric Res.

[111] El-Shazly M (2017). Inhibitory effects of salicylic acid and silver nanoparticles on potato virus Y infected potato plants in Egypt. Middle East J Agric Res.

[112] Rajiv P, Rajeshwari S, Venckatesh R (2013). Bio-fabrication of zinc oxide nanoparticles using leaf extract of *Parthenium hysterophorus* L. and its size-dependent antifungal activity against plant fungal pathogens. Spectrochim Acta A Mol Biomol Spectrosc.

[113] Ogunyemi SO, Zhang M, Abdallah Y, Ahmed T, Qiu W, Ali MA, Yan C, Yang Y, Chen J, Li B (2020). The bio-synthesis of three metal oxide nanoparticles (ZnO, MnO_2_, and MgO) and their antibacterial activity against the bacterial leaf blight pathogen. Front Microbiol.

[114] Dorjee L, Gogoi R, Kamil D, Kumar R, Verma A (2023). Copper nanoparticles hold promise in the effective management of maize diseases without impairing environmental health. Phytoparasitica.

[115] Sadek ME, Shabana YM, Sayed-Ahmed K, Abou Tabl AH (2022). Antifungal activities of sulfur and copper nanoparticles against cucumber postharvest diseases caused by botrytis cinerea and *Sclerotinia sclerotiorum*. J Fungi.

[116] Sathiyabama M, Manikandan A (2018). Application of copper-chitosan nanoparticles stimulate growth and induce resistance in finger millet (*Eleusine coracana* Gaertn) plants against blast disease. J Agric Food Chem.

[117] Vanti GL, Masaphy S, Kurjogi M, Chakrasali S, Nargund VB (2020). Synthesis and application of chitosan-copper nanoparticles on damping off causing plant pathogenic fungi. Int J Biol Macromol.

[118] Tariq M, Mohammad KN, Ahmed B, Siddiqui MA, Lee J (2022). Biological synthesis of silver nanoparticles and prospects in plant disease management. Molecules.

[119] Li Y, Zhang P, Li M, Shakoor N, Adeel M, Zhou P, Guo M, Jiang Y, Zhao W, Lou BZ, Rui Y (2023). Application and mechanisms of metal-based nanoparticles in the control of bacterial and fungal crop diseases. Pest Manag Sci.

[120] Hoang NH, Le Thanh T, Sangpueak R, Thepbandit W, Saengchan C, Papathoti NK, Treekoon J, Kamkaew A, Phansak P, Buensanteai K (2023). The effect of chitosan nanoparticle formulations for control of leaf spot disease on cassava. Phytoparasitica.

[121] Rai M, Shende SS, Paralikar P (2023). In vivo antifungal efficacy of copper and sulfur nanoparticles on soft rot of ginger (*Zingiber Officinale* Rosc.). Bionanoscience.

[122] Khan MR, Rizvi TF (2014). Nanotechnology: scope and application in plant disease management. Plant Pathol J.

[123] Goyal U, Chaubey KK, Pandey SD, Verma DK, Bachheti A, Bachheti RK. Role of gold nanoparticles in plant protection against pathogen; 2024. 201–211.

[124] Du J, Liu B, Zhao T, Xu X, Lin H, Ji Y, Li Y, Li Z, Lu C, Li P, Zhao H, Li Y, Yin Z, Ding X (2022). Silica nanoparticles protect rice against biotic and abiotic stresses. J Nanobiotechnology.

[125] Elmer W, Ma C, White J (2018). Nanoparticles for plant disease management this review comes from a themed issue on nanomaterials in plants. Curr Opin Environ Sci Health.

[126] Safdar M, Kim W, Park S, Gwon Y, Kim YO, Kim J (2022). Engineering plants with carbon nanotubes: a sustainable agriculture approach. J Nanobiotechnol.

[127] Younas A, Yousaf Z, Rashid M, Riaz N, Fiaz S, Aftab A, Haung S. Nanotechnology and plant disease diagnosis and management. Nanoagronomy. 2020: 101–123.

[128] Ahmed T, Luo J, Noman M, Ijaz M, Wang X, Masood HA, Manzoor N, Wang Y, Li B (2023). Microbe-mediated nanoparticle intervention for the management of plant diseases. Crop Health.

[129] Khan MR, Jairajpuri MS. Nematode infestation in food crops-national scenario. Nematode Infestations, Part I: Food Crop; 2010. p. 1–16.

[130] Khan MR, Majid S, Mohidin FA, Khan N (2011). A new bioprocess to produce low cost powder formulations of biocontrol bacteria and fungi to control fusarial wilt and root-knot nematode of pulses. Biol Control.

[131] Xu L, Liu Y, Bai R, Chen C (2010). Applications and toxicological issues surrounding nanotechnology in the food industry. Pure Appl Chem.

[132] Bouwmeester H, Dekkers S, Noordam MY, Hagens WI, Bulder AS, de Heer C, ten Voorde SECG, Wijnhoven SWP, Marvin HJP, Sips AJAM (2009). Review of health safety aspects of nanotechnologies in food production. Regul Toxicol Pharmacol.

[133] Lyons K, Scrinis G. Under the regulatory radar? Nanotechnologies and their impacts for rural Australia; 2009.

[134] Ding Y, Wang Q, Zhu G, Zhang P, Rui Y (2023). Application and perspectives of nanopesticides in agriculture. J Nanoparticle Res.

[135] Priyanka P, Kumar D, Yadav K, Yadav A. Nanopesticides: synthesis, formulation and application in agriculture. Nanotechnol Life Sci. 2019: 129–143.

[136] Fu L, Wang Z, Dhankher OP, Xing B (2020). Nanotechnology as a new sustainable approach for controlling crop diseases and increasing agricultural production. J Exp Bot.

[137] Mousavi SR, Rezaei M (2011). Nanotechnology in agriculture and food production. J Appl Environ Biol Sci.

[138] Kumar S, Kumar D, Dilbaghi N (2017). Preparation, characterization, and bio-efficacy evaluation of controlled release carbendazim-loaded polymeric nanoparticles. Environ Sci Pollut Res Int.

[139] Bhattacharyya A, Duraisamy P, Govindarajan M, Buhroo AA, Prasad R. Nano-biofungicides: emerging trend in insect pest control. 2016: 307–319.

[140] Zhao Y, Zhang Y, Yan Y, Huang Z, Zhang Y, Wang X, Zhou N (2024). PH-responsive pesticide-loaded hollow mesoporous silica nanoparticles with ZnO quantum dots as a gatekeeper for control of rice blast disease. Materials.

